# SLUG Directs the Precursor State of Human Brain Tumor Stem Cells

**DOI:** 10.3390/cancers11111635

**Published:** 2019-10-24

**Authors:** Charles Chesnelong, Xiaoguang Hao, Orsolya Cseh, Alice Yijun Wang, H. Artee Luchman, Samuel Weiss

**Affiliations:** 1 Hotchkiss Brain Institute, Department of Cell Biology and Anatomy, Arnie Charbonneau Cancer Institute, Cumming School of Medicine, University of Calgary, Calgary, AB T2N 4N1, Canada; 2 Arthur and Sonia Labatt Brain Tumor Research Center, Developmental and Stem Cell Biology department, The Hospital for Sick Children, Toronto, ON M5G 0A4, Canada

**Keywords:** glioblastoma (GBM), brain tumor stem cells (BTSC), precursor state, epithelial to mesenchymal transition (EMT), STAT3, SLUG

## Abstract

In glioblastoma (GBM), brain tumor stem cells (BTSCs) encompass heterogenous populations of multipotent, self-renewing, and tumorigenic cells, which have been proposed to be at the root of therapeutic resistance and recurrence. While the functional significance of BTSC heterogeneity remains to be fully determined, we previously distinguished relatively quiescent stem-like precursor state from the more aggressive progenitor-like precursor state. In the present study, we hypothesized that progenitor-like BTSCs arise from stem-like precursors through a mesenchymal transition and drive post-treatment recurrence. We first demonstrate that progenitor-like BTSCs display a more mesenchymal transcriptomic profile. Moreover, we show that both mesenchymal GBMs and progenitor-like BTSCs are characterized by over-activated STAT3/EMT pathways and that SLUG is the primary epithelial to mesenchymal transition (EMT) transcription factor directly regulated by STAT3 in BTSCs. SLUG overexpression in BTSCs enhances invasiveness, promotes inflammation, and shortens survival. Importantly, SLUG overexpression in a quiescent stem-like BTSC line enhances tumorigenesis. Finally, we report that recurrence is associated with SLUG-induced transcriptional changes in both BTSCs and GBM patient samples. Collectively, our findings show that a STAT3-driven precursor state transition, mediated by SLUG, may prime BTSCs to initiate more aggressive mesenchymal recurrence. Targeting the STAT3/SLUG pathway may maintain BTSCs in a quiescent stem-like precursor state, delaying recurrence and improving survival in GBM.

## 1. Introduction

Glioblastoma (GBM) is the most common primary adult brain tumor and is characterized by rapid proliferation, widespread invasion, and aggressive behavior. Despite surgery, radiation, and chemotherapy, GBM invariably recurs resulting in a dismal 5% survival rate at five years [1]. Although yet to be conclusively associated with clinical outcome, transcriptomic studies define four GBM subtypes: proneural, neural, classical, and mesenchymal [2]. While there is a lack of consensus on the frequency and clinical relevance of this phenomenon, a proneural to mesenchymal transition (PMT) has been described in GBM upon recurrence [3].

STAT3 is a pro-survival and pro-inflammatory transcription factor abnormally active in a multitude of cancers, including GBM, where it has emerged as a master regulator of tumorigenesis and a mediator of therapeutic resistance [4]. Importantly, not only is STAT3 one of the key transcription factors linked with the mesenchymal GBM transcriptomic profile, it has also been suggested to be the master regulator of the PMT [3,5]. Moreover, ionizing radiation has been shown to trigger the PMT through STAT3 activation [6,7]. Increasing evidence supports a potential link between PMT and an epithelial to mesenchymal transition (EMT) in GBM. While not classically associated with non-epithelial tumors, an EMT-like process may play a unique role in GBM progression. GBM cells have been proposed to progressively lose glial characteristics and gain mesenchymal features in a so-called mesenchymal drift associated with post-treatment recurrence [8,9]. Interestingly, PMT at recurrence was associated with overactivated STAT3 and increased levels of the key EMT marker and regulator CD44 in matched primary and recurrent GBMs [10]. Moreover, a multi-cancer EMT gene expression signature has been found in non-epithelial cancers, including GBM, where it is associated with shorter time to recurrence post-treatment and strongly correlated with expression of CD44 [11]. Finally, EMT master regulators, such as SNAIL, SLUG, and TWIST, have been shown to promote proliferation and invasion both in vitro and in vivo not only in epithelial cancers but also in GBM [12,13,14].

The discovery of adult neural stem cells in the mammalian brain [15] has paved the way for the identification of cancer stem cells in GBM; the brain tumor stem cells (BTSCs). The extensive self-renewal potential, proliferative capacity, and therapeutic resistance of BTSCs, as well as their ability to phenocopy parent tumors in mice, suggest that they are integral to the growth and post-treatment recurrence of GBM [16,17,18,19]. Hence, BTSCs represent a source of disease that needs to be specifically targeted if GBM outcome is to be improved. Our laboratory has successfully established BTSC lines from more than 150 GBM patients. These BTSCs are representative of the molecular heterogeneity of the disease as they include subsets with mutations representing the most prevalent alterations in human GBM [20,21,22]. Most importantly, xenografts of BTSC lines in mice provide an improved in vivo GBM model recapitulating the diffuse invasion and necrosis patterns of human GBM [20,21]. The experimental therapeutic value of BTSCs is exemplified by our identification of a dual mTORC1/2 inhibitor for GBM [23], which has led to a recently completed NCIC-CTG Phase I/II clinical trial.

While there is consensus that BTSCs need to be specifically targeted to improve GBM outcome, a major challenge remains the lack of understanding of BTSC heterogeneity. We previously studied heterogeneity in a large set of BTSC cultures and identified two groups with distinct precursor states [24]. Stem-like BTSCs express the stem cell marker CD133, have a higher fraction of quiescent cells, and divide asymmetrically, while progenitor-like BTSCs are characterized by faster proliferation, expression of neural progenitor markers, and strikingly shorter survival in orthotopic xenografts. Interestingly, differentially expressed genes between stem- and progenitor-like BTSCs also segregate proneural from mesenchymal GBM samples. In this study, a unique stem/progenitor nomenclature was judiciously chosen to label these two distinct BTSC groups. This classification, which reflects the cancer stem cell dogma, is based on the careful phenotypic characterization of a large collection of BTSCs. Several other groups have reported two distinct groups of BTSCs that, while not specifically using the stem/progenitor nomenclature, show striking resemblance to our stem-like and progenitor-like groups. These studies indeed report BTSC groups resembling either the proneural or mesenchymal GBM subtypes. In these studies, mesenchymal BTSCs have smaller CD133+, and larger CD44+, subpopulations and present a more glycolytic, radio-resistant, and aggressive phenotype both in vitro and in vivo [25,26,27]. Investigating whether proneural and mesenchymal transcriptomic profiles are associated with stem-like and progenitor-like BTSC precursor states is essential to confirm the existence of consolidated proneural/stem-like and mesenchymal/progenitor-like BTSC groups.

The identification of distinct BTSCs precursor states has introduced a new perspective and a better understanding of these cells. One could reason that proneural/stem-like and mesenchymal/progenitor-like BTSCs represent separate entities, which would thus need to be studied independently. Alternatively, a hierarchy may exist and it becomes reasonable to propose that specific molecular pathways regulate a transition from stem-like to progenitor-like BTSCs. The idea of a hierarchy between stem-like and progenitor-like BTSCs comes from NSC biology and fits within the CSC hypothesis. Furthermore, the common proneural origin of proneural and mesenchymal GBM subtypes also suggest such hierarchy even in the bulk of the tumor [28]. Confirming whether a hierarchical relationship exists between these two groups is key to the better understanding of glioma stem cell biology.

With the premise that a hierarchical paradigm underlies these groups, we hypothesized that the mesenchymal/progenitor-like BTSCs arise from a less aggressive, proneural/stem-like precursor through an EMT-like, STAT3-driven transition. In this study, we demonstrate that a STAT3/SLUG-driven process promotes a more aggressive progenitor-like precursor state in BTSCs and that this transition is associated with recurrence in GBM. Our findings suggest that a STAT3/SLUG-driven precursor state transition may be triggered upon treatment in the CSC compartment and represent the initial step to recurrence. Therapeutic strategies aimed at targeting this novel STAT3/SLUG pathway may prove particularly effective at blocking this transition, prevent or delay recurrence, and ultimately significantly improve survival of GBM patients.

## 2. Results

### 2.1. Progenitor-Like BTSCs Display a Mesenchymal Expression Profile Associated with Over-Activated STAT3 and EMT Pathways

**Figure 1:**
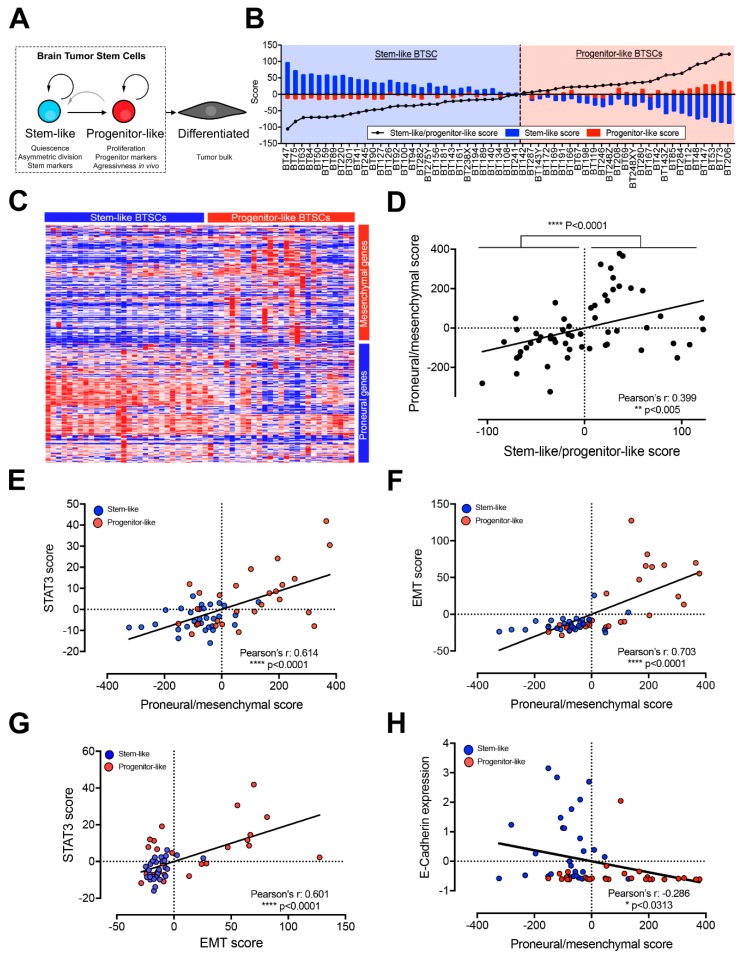
Stem-like and progenitor-like brain tumor stem cells (BTSCs) display proneural and mesenchymal transcriptomic profiles respectively, which correlate with epithelial to mesenchymal transition (EMT) and STAT3 pathways’ activity (see also Figures S1–S3 and Table S1). (**A**) Schematic representation of BTSC precursor states. (**B**) Bar graph showing our cohort of 57 BTSCs ranked based on the stem-like to progenitor-like score and segregated as stem-like (30 BTSCs) or progenitor-like (27 BTSCs). (**C**) Heatmap representing the expression of proneural and mesenchymal genes (from [2] and listed in Table S1) in stem-like and progenitor-like BTSCs in the same order than Figure 1B (expression data can be found on the EGA platform). (**D**) Scatter plot showing the correlation between the proneural to mesenchymal and the stem-like to progenitor-like scores in BTSCS. Scatter plots showing the correlation between the proneural to mesenchymal score and (**E**) STAT3 or (**F**) EMT scores in BTSCs segregated as stem-like (blue) or progenitor-like (red). Scatter plot showing (**G**) the correlation of STAT3 and EMT scores and (**H**) the correlation between the proneural to mesenchymal score and E-cadherin expression in BTSCs segregated as stem-like (blue) or progenitor-like (red). All figures are based on RNA-sequencing performed on 57 BTSC lines.

We previously used classic neural stem cell characteristics to define distinct stem-like and progenitor-like precursor states in BTSCs (schematic representation in Figure 1A) [24]. In this study we found that differentially expressed genes between these two groups segregate proneural from mesenchymal GBMs. Here, we first confirm that the stem-like to progenitor-like score does correlate with the proneural to mesenchymal score of GBMs tissue samples from all transcriptomic subtypes (Figure S1A). Normal brain tissue and proneural GBMs have, as expected, low stem-like to progenitor-like scores while mesenchymal GBMs are characterized by high stem-like to progenitor-like scores (Figure S1B). Interestingly, the proneural to mesenchymal score also correlates with the stem-like to progenitor-like score in classical and neural GBM subtypes (Figure S1C,D). This finding illustrates that high mesenchymal/progenitor-like signatures can be found in GBMs from all four transcriptomic subtypes and thus suggests that the precursor state association with GBM transcriptomic subtypes may not be limited to proneural and mesenchymal GBMs.

Following the above validation of our previous findings, we asked whether, conversely, stem-like and progenitor-like BTSCs display proneural and mesenchymal transcriptomic profiles, respectively. Using RNA-sequencing, we ranked and segregated 57 BTSC lines according to their stem-like to progenitor-like score (Figure 1B and Table S1). We observed a higher expression of proneural genes in stem-like BTSCs and of mesenchymal genes in progenitor-like BTSCs (Figure 1C) [2] and overall a significant correlation between the proneural to mesenchymal and stem-like to progenitor-like scores (Figure S1D).

Based on these findings and the premise that a hierarchical paradigm underlies these groups, we hypothesized that mesenchymal/progenitor-like BTSCs arise from a less aggressive proneural/stem-like precursor. We further surmized that this phenomenon arises through a STAT3-driven mesenchymal transition in the CSC compartment and is at the root of the PMT observed at recurrence in GBM [3]. To validate the premise of this hypothesis, we calculated STAT3 and EMT scores (Table S1) [11] and noted a striking correlation in TCGA GBM samples (Figure S2A). As expected, samples with high STAT3 and EMT scores are enriched in mesenchymal GBMs (Figure S2B). Conversely, samples with low STAT3 and EMT scores are enriched in normal brain tissue and proneural GBMs (Figure S2B). These findings confirm that STAT3 and EMT pathways are both overactivated in mesenchymal GBMs. Interestingly, a correlation between STAT3 and EMT scores also exist among neural and classical GBMs (Figure S2C,D). A putative STAT3-driven mesenchymal transition may thus be relevant to all GBMs independently of their respective transcriptomic subgroups. Supporting the clinical relevance of such a pathway in GBM, STAT3^+^/EMT^+^ GBM patients have significantly shorter survival than STAT3^−^/EMT^−^ GBMs (Figure S2E,F).

We next investigated this same pathway in BTSCs and similarly observed a significant correlation between the proneural to mesenchymal score and the STAT3 and EMT scores (Figure 1E,F). STAT3 and EMT scores are both significantly higher in progenitor-like than in stem-like BTSCs (Figure S3A,B). Interestingly, while the stem-like to progenitor-like score significantly correlates with the STAT3 score, the correlation with the EMT score did not quite reach significance because of a subgroup of progenitor BTSCs presenting a lower EMT score. However, STAT3 and EMT scores are significantly correlated to each other in BTSCs, and progenitor-like BTSCs are enriched among samples with high STAT3 and EMT scores (Figure 1G). Given that E-cadherin repression is the central event defining the EMT process, we then examined E-cadherin expression and found an inverse correlation with the proneural to mesenchymal score and significantly lower expression levels in progenitor-like compared to stem-like BTSCs (Figure 1H and Figure S3C). Taken together, these results suggest that a STAT3-driven EMT-like process may play a role in promoting the aggressive progenitor-like BTSC precursor state and may be relevant to the PMT.

### 2.2. EMT Master Regulators Are Enriched in Progenitor-Like BTSCs

**Figure 2:**
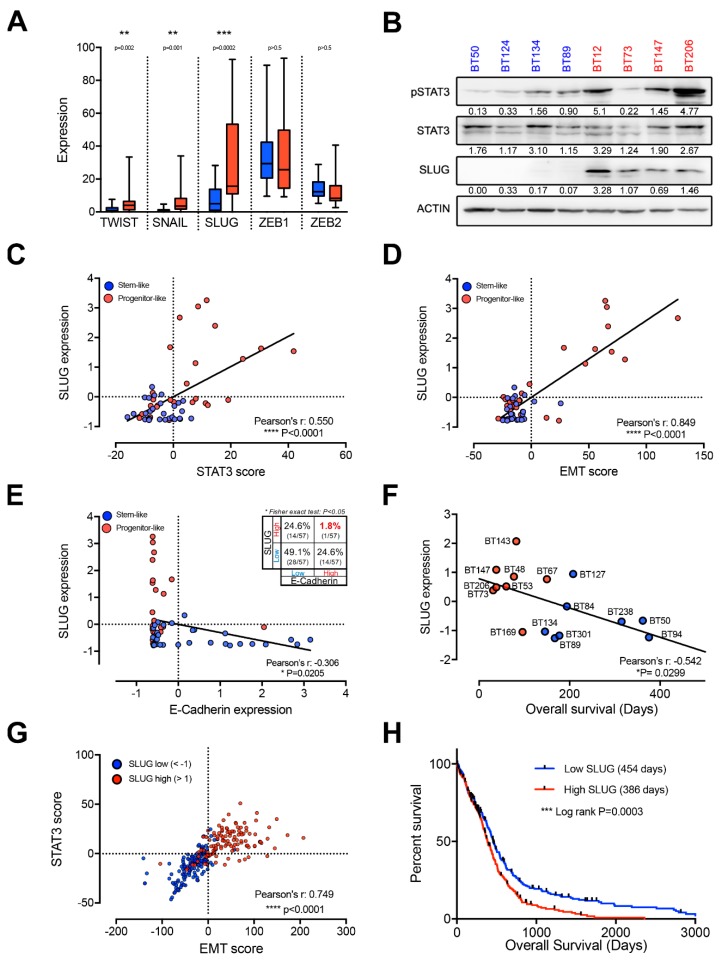
SLUG is the key EMT transcription factor in progenitor-like BTSCs (see also Figure S4). (**A**) Box and whiskers (min to max) plot of the expression of EMT master regulators in stem-like (blue) and progenitor-like (red) BTSCs. (**B**) Representative western blot showing increased activated STAT3 (pSTAT3-Y705) and higher SLUG levels in progenitor-like (red) compared to stem-like (blue) BTSCs. Quantification relative to loading controls. Scatter plots representing the correlation between SLUG expression and (**C**) STAT3 and (**D**) EMT scores. (**E**) Scatter plots showing the mutually exclusive expression of SLUG with E-cadherin in BTSCs segregated as stem-like (blue) or progenitor-like (red). (**F**) Scatter plot illustrating the inverse correlation between expression of SLUG and available BTSC survival data. (**G**) Scatter plot representing the correlation between STAT3 and EMT scores in SLUG^high^ (red, z-score < 1) and SLUG^low^ (blue, z-score >1) TCGA GBM (glioblastoma) samples (Affymetrix U133a microarray platform). (**H**) Kaplan Meier survival curves from 523 GBM samples segregated around the geometric mean of SLUG expression. 256 samples were below (Low SLUG, in blue, 454 days) and 267 samples above (High SLUG, in red, 386 days) this geometric mean. Expression data is from TCGA Affymetrix U133a microarray platform. Box and whiskers plot in (**A**), scatter plots (**C**–**E**) and SLUG expression in (**F**) are based on RNA-sequencing performed on 57 BTSC lines.

We next asked which transcription factors may be driving this putative EMT-like process. Surprisingly, ZEB1 and ZEB2 are not differentially expressed in stem-like and progenitor-like BTSCs (Figure 2A) and do not show strong positive correlation with STAT3 or EMT scores (Figure S4A–D). However, we found significantly higher expression of the other three key EMT regulators, TWIST, SNAIL, and SLUG, in progenitor-like BTSCs (Figure 2A). Interestingly, SLUG is the most highly expressed of these factors and shows the most significant differential expression between stem-like and progenitor-like BTSCs (Figure 2A). Moreover, we observed strikingly higher protein levels of SLUG and activated STAT3 (phosphorylated Y705) in progenitor-like BTSCs compared to stem-like BTSC (Figure 2B). Furthermore, SLUG shows the best correlation with both STAT3 and EMT scores (Figure 2C,D and Figure S4E–H). As mentionned above, loss of E-cadherin is the central event that defines EMT at the molecular level and SLUG as a key EMT factor is a repressor of E-cadherin. Thus, the expression of SLUG and E-cadherin is expected to be inversely correlated. Here, we show that SLUG and E-cadherin are not only inversely correlated (r = −0.306) but also mutually exclusive (Fisher’s exact test *p* > 0.05; Figure 2E) suggesting that SLUG may regulate an EMT-like process in GBM. Collectively, these findings suggest that SLUG is a key factor in the putative STAT3-driven, EMT-like process in progenitor-like BTSCs.

To evaluate whether SLUG may play a role in the aggressive nature of progenitor-like BTSCs, we correlated its expression in several BTSCs with survival data from orthotopic xenografts of these cells. Strikingly, SLUG expression correlates with shorter survival time in xenografts (Figure 2F). Importantly, SLUG expression also correlates with STAT3 and EMT scores in GBMs and predicts shorter patient survival (Figure 2G,H).

### 2.3. SLUG Is the Main EMT Master Regulator Directly Regulated by STAT3 in BTSCs

**Figure 3:**
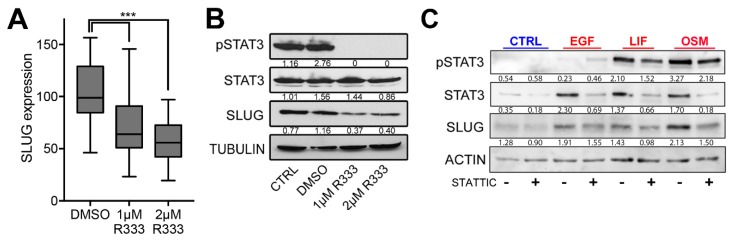

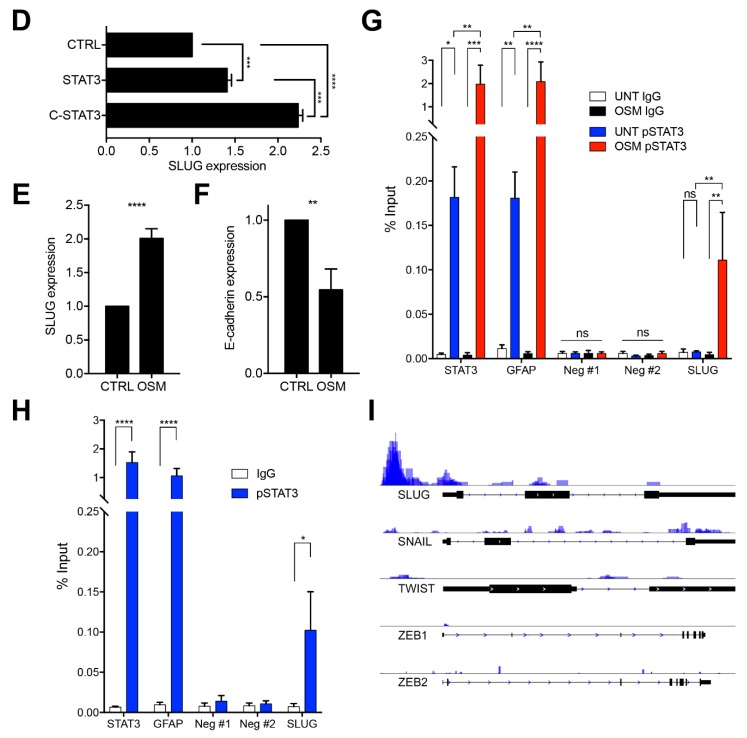
SLUG is the primary direct transcriptional target of STAT3 in BTSC (see also Figure S5). (**A**) Box and whiskers graph (min to max) representing SLUG expression measured by qPCR in 12 BTSCs (as detailed in Section 4.1) treated with JAK3 inhibitor (R333) and (**B**) representative western blot quantified relative to loading control. (**C**) Representative western blot (with quantification values relative to loading control) showing activated STAT3 (pSTAT3-Y705) and SLUG protein levels in BT67 24 h post epidermal growth factor (EGF), leukemia inibitory factor (LIF), and oncostatin M (OSM) treatments with or without concurrent inhibition of STAT3 with direct STAT3 inhibitor STATTIC (10 µM). (**D**) Bar graph of SLUG expression measured by qPCR in empty vector, wild-type STAT3, or constitutive STAT3, overexpressing BT124. RT-qPCR data representing (**E**) SLUG and (**F**) E-cadherin expression following OSM treatment (10 ng/mL) in BT67. Positive controls (STAT3 and GFAP), negative controls and SLUG enrichment results from ChIP-PCR experiments performed (**G**) on BT67 in growth factor-free media with or without OSM or (**H**) on BT50, BT67, BT69, BT89, BT94, BT147 grown in standard BTSC culture condition (+EGF, +FGF). (**I**) Histograms representing binding of STAT3 on the promoter of SLUG, SNAIL, TWIST, ZEB1, and ZEB2 from whole genome ChIP-seq experiments performed on the same 6 BTSC lines (BT50, BT67, BT69, BT89, BT94, BT147) maintained in standard BTSC culture condition (+EGF, +FGF). (**D**–**H**), SEM, ns. p>0.05; * *p* < 0.05; ** *p* < 0.01; *** *p* < 0.001; **** *p* < 0.0001.

We then asked whether STAT3 regulates SLUG expression in BTSCs. Inhibition of the STAT3 pathway with a Janus Kinase 3 (JAK3) inhibitor (R333) [29] decreased the expression of SLUG in 12 BTSC lines (Figure 3A). Downregulation of SLUG was also observed at the protein level (Figure 3B). Using a JAK2 inhibitor, SB1518 [30], and STAT3 SH2 domain inhibitors, such as SH-04-54 [31] or STATTIC (STAT three inhibitory compound) [32], we further confirmed that SLUG is downregulated upon STAT3 inhibition (Figure S5A,B).

We next stimulated the STAT3 pathway with epidermal growth factor (EGF), leukemia inibitory factor (LIF), or oncostatin M (OSM). All treatments triggered SLUG upregulation that could be blocked upon concurrent treatment with STATTIC (Figure 3C and Figure S5E). Overexpression of STAT3 in a stem-like BTSC line with low basal levels of activated STAT3 (BT124), also triggered a significant upregulation of SLUG, which was further increased when overexpressing a constitutively active form of STAT3 (Figure 3D).

To determine whether STAT3 regulates SLUG directly, we performed a transcription factor binding site search and found a potential binding site, which we proceeded to validate through chromatin immunoprecipitation (ChIP). OSM is an extremely potent activator of STAT3 signaling in BTSCs and extremely low doses are sufficient to activate STAT3 and increase SLUG levels (Figure 3C and Figure S5C). We treated cells (BT67) with 10 ng/mL of OSM, which not only triggered a robust increase in SLUG expression (Figure 3E) but also lead to significant repression of E-cadherin expression (Figure 3F). Following OSM-induced activation of STAT3 in cells otherwise maintained in growth factor-depleted media, a specific and significant enrichment of SLUG is observed, demonstrating that STAT3 directly regulates SLUG (Figure 3G and Figure S5D).

BTSCs are grown in serum-free media supplemented with EGF and FGF, which are both potent STAT3 activators. ChIP performed on 6 BTSC lines (BT50, BT67, BT69, BT89, BT94, BT147) maintained under these conditions, showed binding of STAT3 to the SLUG promoter (Figure 3H). Interestingly, this was not observed in absence of growth factors (Figure 3G, untreated condition). We also performed whole genome ChIP-sequencing on the same six BTSC lines cultured in standard growth factor-supplemented media and confirmed the binding of STAT3 to the SLUG promoter. Surprisingly, STAT3 did not appear to directly regulate TWIST, a well characterized transcriptional target of STAT3, nor SNAIL, ZEB1, or ZEB2 (Figure 3I). These results demonstrate that SLUG is not only a transcriptional target of STAT3 but, most importantly, the only EMT transcription factor directly regulated by STAT3 in BTSCs. SLUG is thus the most likely mediator of a putative STAT3-driven EMT-like process toward the BTSC progenitor-like precursor state.

### 2.4. SLUG Overexpression Promotes BTSC Migration and Invasion Both In Vitro and In Vivo

**Figure 4:**
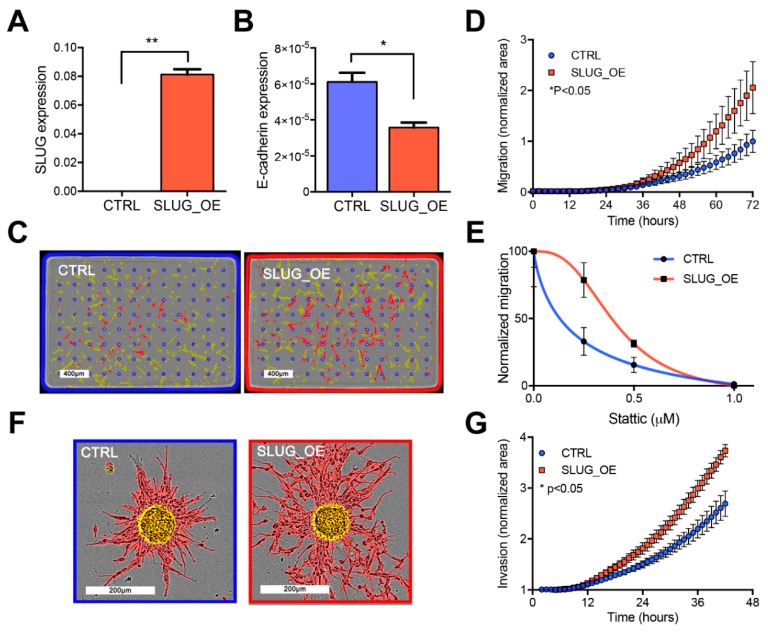

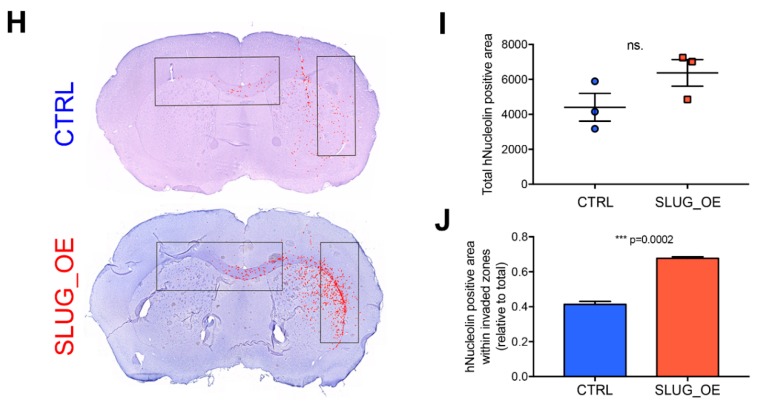
SLUG promotes migration and invasion both in vitro and in vivo (see also Figure S6). Bar graphs representing (**A**) SLUG and (**B**) E-cadherin expression in BT69 control (CTRL) and overexpressing SLUG (SLUG_OE) as measured by RT-PCR (* *p* < 0.05; ** *p* < 0.01). (**C**) Representative images of cells on the upper side of the membrane (highlighted in yellow) and cells that have migrated to the bottom side (highlighted in red) and (**D**) quantified migration of CTRL and SLUG_OE cells. (**E**) Graph of STAT3 inhibition (STATTIC) impact on migration of BT69 CTRL and SLUG_OE. (**F**) Representative overlaid images of spheres embedded in collagen at T0 (highlighted in yellow) and pictures of invaded cells at end point (highlighted in red). (**G**) Graphical representation of the quantified invasion of BT69 CTRL and SLUG_OE cells in vitro (area of invaded cells (highlighted in red) was normalized to the area of the imbedded spheres at T0 (highlighted in yellow). (**H**) Representative pictures of the invasive behavior of BT69 CTRL and SLUG_OE cells in a corpus callosum implantation assay. hNucl positive cells were overlaid with large red dots on the high resolution images to illustrate the tumor cell distribution throughout the brain at endpoint. (**I**) Total hNucl positive area throughout the brain sections. (**J**) Graphical representation of the hNucl positive area in invaded zones (represented as rectangles) normalized to total hNucl positive area. Error bars represent SEM (**A**–**J**).

We next asked whether SLUG is involved in the more aggressive phenotype of progenitor-like BTSCs. We stably overexpressed SLUG in BT69, which has low endogenous expression of SLUG and confirmed that SLUG overexpression (SLUG_OE; Figure 4A) triggers downregulation of E-cadherin (Figure 4B). Increased motility is the main characteristic of cells undergoing EMT. We thus performed real-time chemotaxis assays and observed significantly increased migration of SLUG_OE cells as compared to their matched controls (CTRL; Figure 4C,D). Overexpression of SLUG in additional BTSC lines similarly triggered enhanced migration potential (Figure S6A–C). Interestingly, overexpression of SLUG partially rescued the inhibitory effect of STATTIC on migration, suggesting that SLUG mediates the migration enhancing roles of STAT3 in BTSCs (Figure 4E). In addition to enhanced migration, SLUG_OE cells also displayed a significantly increased invasive behavior in a collagen matrix (Figure 4F,G).

We next asked whether SLUG overexpression promotes BTSC invasion in vivo. Given the paucity of reliable in vivo invasion assays, we developed a corpus callosum invasion assay that makes use of the propensity for brain tumor cells to invade along white matter tracts [33]. This assay is essential to evaluate the in vivo invasion potential of cells that do not quickly form massive tumors such as stem-like BTSCs, which are charaterized by extremely slow growth in vivo and long survival (Figure 2F). This technique allows for the assesement of the invasion potential of such cells at relatively short time-points and facilitates the detection of the tumor cells, which can easily be found along the white matter tracts of the corpus callosum. Invasion of CTRL or SLUG_OE cells was quantified 4 weeks post-implantation in designated zones along the corpus callosum and away from the implantation site (Figure 4H). Although we notice an increased growth in SLUG-OE implanted mice, it was not statistically significant (Figure 4H,I). However, SLUG_OE tumors demonstrated significantly increased invasive behavior in vivo (Figure 4H,J).

### 2.5. SLUG Overexpression Leads to Shorter Survival In Vivo

**Figure 5:**
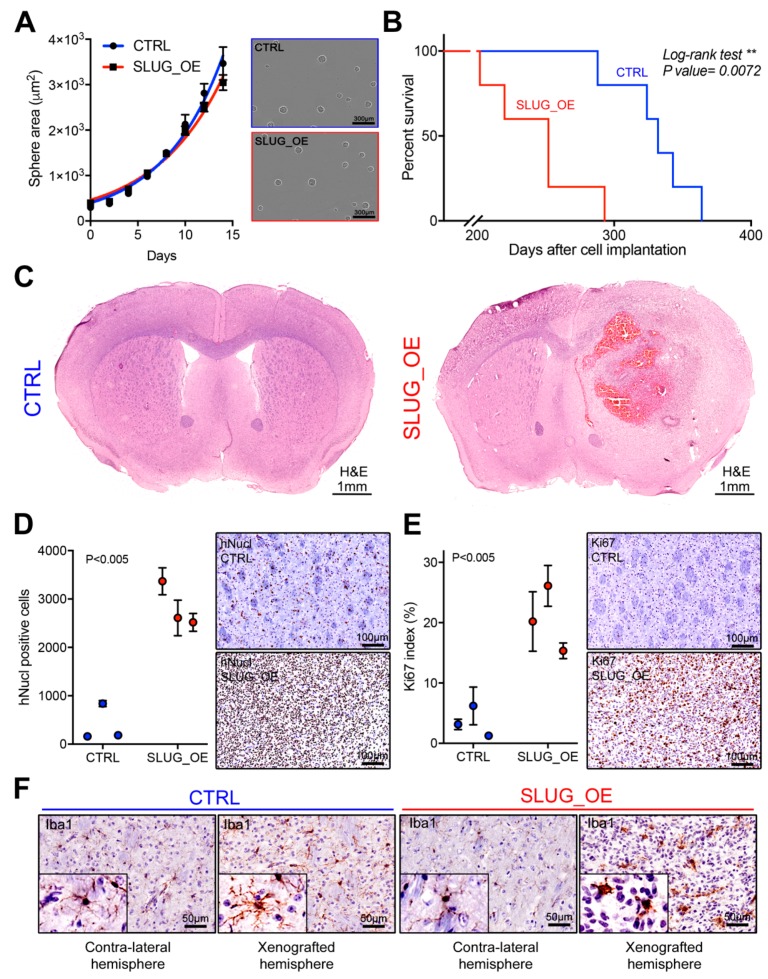
SLUG overexpression promotes a more proliferative, inflammatory, and aggressive phenotype in vivo (see also Figure S6). (**A**) Graphical representation of BT69 CTRL and SLUG_OE sphere size over time and representative images at day 10. (**B**) Kaplan–Meier curves of mice xenografted with BT69 CTRL and SLUG_OE cells. (**C**) Representative H&E sections of BT69 CTRL and SLUG_OE tumors. Quantitative analysis of (**D**) hNucl positive cells, (**E**) Ki67 index in CTRL and SLUG_OE tumors and representative images. (**F**) Representative images with 2.5x close-up inserts of Iba1 staining in the xenografted and contra-lateral hemispheres of mice xenografted with CTRL and SLUG_OE cells. Error bars represent SD (**A**,**D**,**E**).

SLUG overexpression was previously shown to promote proliferation in multiple cancer cell lines, including GBM [14]. However, SLUG overexpression did not result in increased BTSC growth in vitro (Figure 5A and Figure S6D–F). Similarly, overexpression of SLUG did not impact sphere forming frequency (Figure S7A), growth dependency on growth factors (Figure S7B), or growth sensitivity to STAT3 inhibition (Figure S7C).

The lack of impact of SLUG overexpression on BTSC growth in vitro was somewhat surprising. Nonetheless, we asked whether SLUG overexpression could promote tumor growth in an in vivo setting. CTRL or SLUG_OE cells were implanted orthotopically in the striatum of 6- to 8-week-old female C17/SCID mice (n = 7). Strikingly, mice xenografted with SLUG_OE cells showed significantly shorter survival than CTRL (Figure 5B). Moreover, histological analysis of the brains of CTRL animals did not reveal obvious tumor masses based on H&E staining but large aggressive tumors were observed in SLUG_OE injected mice (Figure 5C). Interestingly, while SLUG_OE mice had significantly higher numbers of tumor cells in the injected hemisphere (hNucl staining), CTRL cells were also detected and diffusely present throughout the hemisphere (Figure 5D). We further confirmed that SLUG_OE tumors were strikingly more proliferative, as indicated by a significantly higher Ki67 index (Figure 5E). Finally, we performed immunostaining for the microglia/macrophage-specific protein Iba1, to examine whether there was an increased inflammatory component in the SLUG_OE tumors. Interestingly, while resting ramified microglia are observed in CTRL tumors, and in the contra-lateral hemisphere of BT69 SLUG_OE brain, the SLUG_OE tumor predominantly displays amoeboid phagocytic microglia/macrophages, indicative of a highly inflammatory tumor microenvironment (Figure 5F). Thus, in contrast to the results obtained in vitro, we show here that SLUG overexpression promotes BTSC growth in vivo and leads to larger, more aggressive and inflammatory tumors associated with significantly shorter survival.

### 2.6. SLUG Drives a Shift toward an Aggressive Progenitor-Like Precursor State

**Figure 6:**
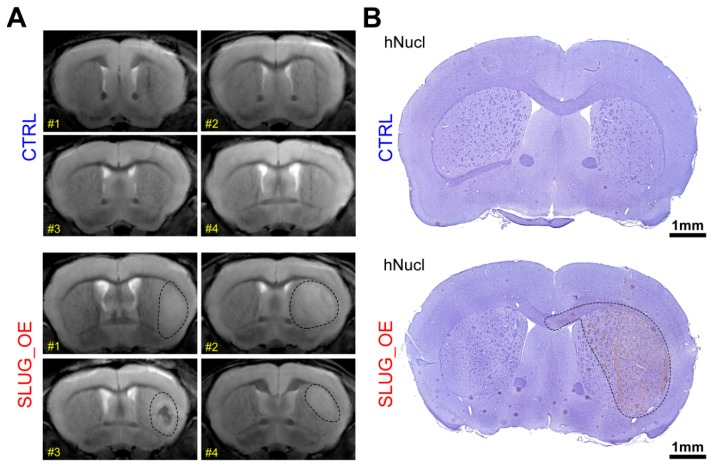

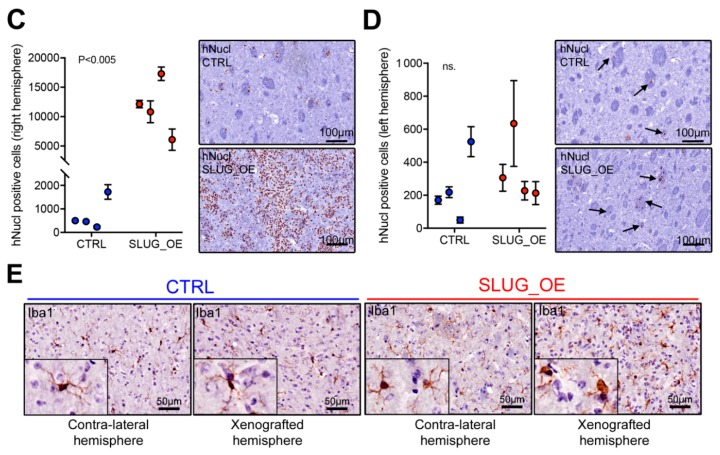
SLUG overexpression in a quiescent stem-like BTSC line promotes tumor initiation in vivo (see also Figure S8). (**A**) Brain MRI of mice a year post-xenograft with BT50 CTRL or SLUG_OE cells (dashed lines represent approximate delimitation of enhancing regions) and (**B**) representative hNucl staining (dashed line represents an approximate tumor delineation). Quantitative analysis and representative close-up hNucl staining of (**C**) the implanted (right) hemisphere and (**D**) the contralateral (left) hemisphere (error bars represent SD). (**E**) Representative images and 2.5× close-up inserts of Iba1 staining in the xenografted and contra-lateral hemispheres of CTRL and SLUG_OE xenografted mice.

To evaluate whether SLUG promotes a transition toward a more progenitor-like precursor state, we overexpressed SLUG in one of our most stem-like BTSC lines (BT50). We then xenografted these BT50 SLUG_OE and CTRL cells in the striatum of 6- to 8-week-old female C17/SCID mice and monitored the animals by MRI. A year post-xenograft, all BT50 SLUG_OE mice started to present large enhancing regions in the xenografted hemisphere and one mouse displayed a large hematoma/necrosis-like feature (BT50 SLUG_OE mice #3). Remarkably, MRI did not reveal any particular features in the xenografted hemisphere of BT50 CTRL mice other than the needle tract that remained visible (Figure 6A). These MRI images strongly suggest development of tumors in BT50 SLUG_OE but not BT50 CTRL mice. As these mice were likely to start developing age-related symptoms unrelated to brain tumors after this one year post-xenograft time point, we elected not to perform a Kaplan Meier survival study and rather chose to focus on this earlier phase of tumor development. All of the study animals were thus sacrificed for further histological analysis. Immuno-histological hNucl staining confirmed the absence of tumors in CTRL animals and the presence of large infiltrative tumors in SLUG_OE mice (Figure 6B). While a significantly higher number of hNucl positive cells are present in the injected hemisphere of SLUG_OE animals, compared to mice implanted with CTRL cells (Figure 6C), we did not observe increased invasion toward the contralateral side (Figure 6D). Nonetheless, these results show that SLUG overexpression increased the tumorigenic potential of this extremely slow growing, quiescent stem-like BTSC line. Strikingly, as observed in BT69 SLUG_OE tumors, BT50 SLUG_OE tumors also present activated phagocytic microglia/macrophages, while ramified microglia are observed in the contralateral hemisphere and throughout the CTRL tumors (Figure 6E).

### 2.7. SLUG-Induced Transcriptional Changes Associate with Progenitor-Like BTSC Precursor State and Recurrence

**Figure 7:**
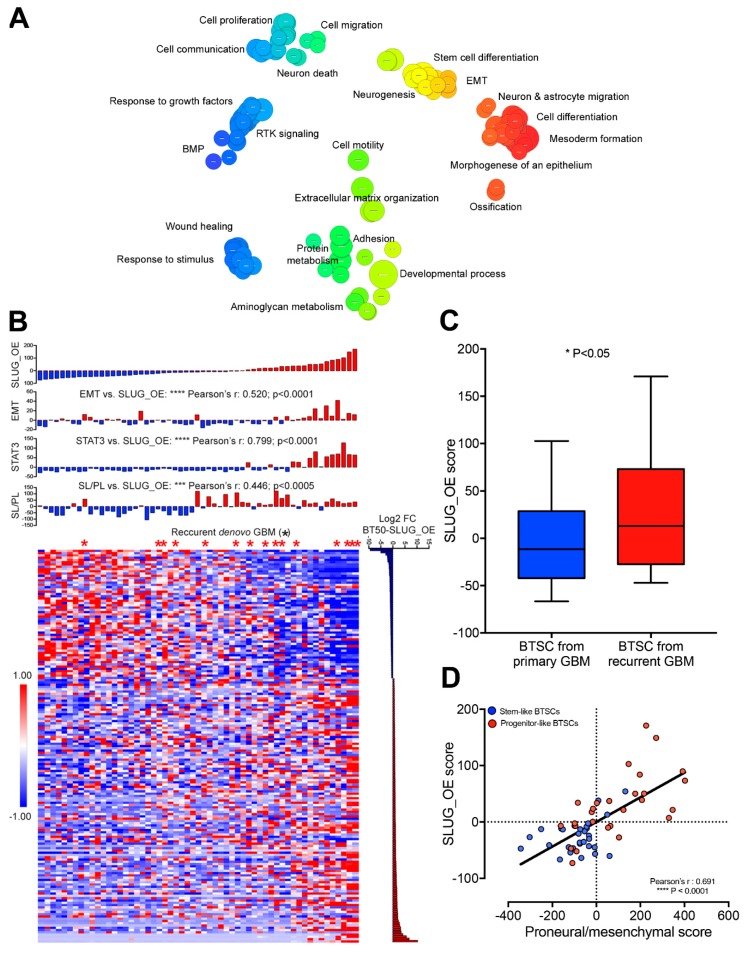
SLUG-induced transcriptional changes are associated with progenitor-like BTSC precursor state and recurrence (see also Table S2). (**A**) Revigo visualization of Gene Ontology analysis from RNA sequencing performed on BT50 CTRL and SLUG_OE cells. (**B**) Heatmap of the 180 differentially expressed genes between BT50 CTRL and SLUG_OE cells in the 57 BTSCs and bar graphs of their respective SLUG_OE, stem-like to progenitor-like (SL/PL), STAT3, and EMT scores (asterisk mark BTSCs from recurrent primary GBMs). (**C**) Box and whiskers plot (min to max) representing the SLUG_OE score of BTSCs that were derived from primary GBMs compared to those derived from recurrent GBMs (exclusively from de novo GBMs). (**D**) Scatter plot of SLUG_OE score versus proneural to mesenchymal score.

We next asked whether the SLUG-induced tumorigenicity of a quiescent stem-like BTSC line is mediated by a transcriptomic shift, underlying a transition toward a progenitor-like precursor state. First, RNA sequencing of BT50 CTRL and SLUG_OE cells from in vitro studies was used to identify 180 differentially expressed genes. Next, Gene Ontology (GO) analysis demonstrated enrichment of GO terms such as proliferation, adhesion, migration, differentiation, neurogenesis, and EMT, upon SLUG overexpression (Figure 7A and Table S2). We then calculated a SLUG_OE score to be used as a proxy of the transcriptional changes induced upon SLUG overexpression in this stem-like BTSC line. Interestingly, the BTSC SLUG_OE score correlates significantly with the stem-like to progenitor-like score (Figure 7B). This indicates that SLUG overexpression may push BTSCs towards a more aggressive phenotype, by promoting a transcriptional transition from the stem-like to progenitor-like precursor state. STAT3 and EMT scores were also correlated with the SLUG_OE score suggesting that a STAT3-driven EMT-like process is regulating this transition (Figure 7B). Remarkably, we observed that BTSC lines derived from recurrent de novo GBMs are enriched among high SLUG_OE score lines (Figure 7B). The SLUG_OE score was indeed found to be significantly higher in BTSCs derived from recurrent de novo GBMs than in all other BTSCs (Figure 7C). Interestingly, the SLUG_OE score of BTSC lines showed a strikingly strong correlation with the proneural to mesenchymal score, supporting our hypothesis that STAT3/SLUG-driven precursor state transition may play an important role in mesenchymal recurrence (Figure 7D).

We further asked whether a shift toward a progenitor-like precursor state underlies the progression from primary to recurrent tumors in GBM patients. We used RNA sequencing data from our only matched pair of primary/recurrent tumors and succesfully derived BTSCs, to examine transcriptomic changes arising upon recurrence. We found EMT and STAT3 scores to be increased in the tumor and its derived BTSC upon recurrence (Figure S8A). We also observed, upon recurrence, increased proneural to mesenchymal score in the tumor and increased stem/progenitor-like and SLUG_OE score in the derived BTSC culture (Figure S8B,C).

**Figure 8:**
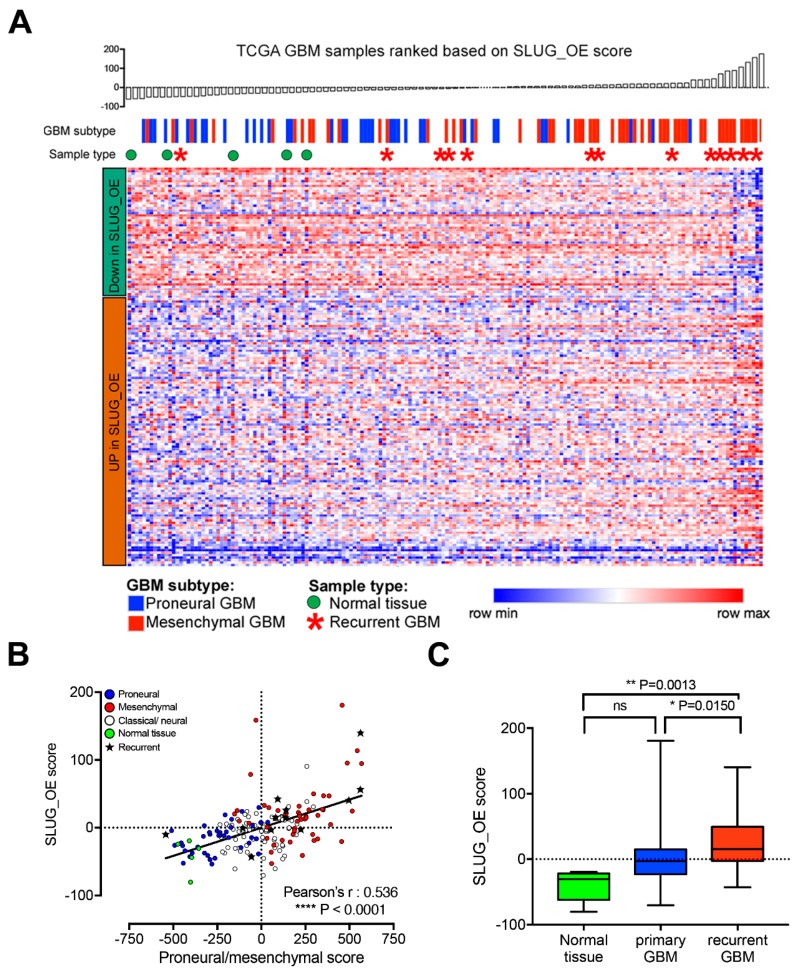
SLUG-induced transcriptional changes are associated with recurrence in GBM patients. (**A**) Heatmap representing the expression of the 180 genes of the SLUG_OE score in 172 TCGA GBM samples. Samples were ranked according to the SLUG_OE score. (**B**) Scatter plot showing correlation between the SLUG_OE score and the proneural to mesenchymal score and association with recurrent samples (annotated with a star). (**C**) Box and whiskers plot (min to max) showing increased SLUG_OE score in recurrent compared primary GBMs and normal brain tissue samples.

Although it would have been optimal to investigate this phenomenon in additional matched pairs of primary/recurrent tumors and their derived BTSCs, these are extremely rare. Similarly, studies on matched primary and recurrent GBM are scarce [10,34] and only limited transcriptomic data is publicly available. Thus, to further assess a potential association of the SLUG_OE score with recurrent mesenchymal GBM, we ranked TCGA GBM samples according to our SLUG_OE score. As expected, the SLUG_OE score correlates significantly with proneural and mesenchymal samples (Figure 8A,B). However, more importantly, we observed that recurrent GBMs are enriched among samples with higher SLUG_OE scores (Figure 8A,B). Further analyses showed significantly increased SLUG_OE score in recurrent GBM compared to primary GBMs and normal brain tissue samples (Figure 8C). Taken together with the rest of this study, these data suggest that a STAT3-driven EMT-like process, mediated by SLUG, drives a precursor state shift that is clinically relevant and may play an essential role in GBM recurrence.

## 3. Discussion

GBM transcriptomic subtypes and particularly the proneural and mesenchymal groups offer solid sub-classification of GBMs. However, they have yet to show biological and clinical relevance. We demonstrate here that stem-like and progenitor-like BTSCs present proneural and mesenchymal transcriptomic profiles, respectively. While an inflammatory microenvironment is undoubtedly associated with, and contributes to, the transcriptomic profile of mesenchymal GBMs, our results suggests that mesenchymal GBMs may originate from a subpopulation of proneural stem-like BTSCs that have acquired a more mesenchymal/progenitor-like precursor state through an EMT-like process. We also propose that a putative STAT3-driven mesenchymal/progenitor-like transition is not only relevant to proneural and mesenchymal GBMs, although they may exemplify extreme cases, but also to classical and neural GBMs. It is noteworthy that the classical transcriptomic subtype may largely reflect alterations in RTKs and cell cycle regulators and the neural subtype is disappearing altogether from the classification [35]. Importantly, while EMT is a dedifferentiation process generating cancer stem cells in epithelial cancers [36], a similar signaling pathway may underlie a distinct process in the cancer stem cell compartment of non-epithelial tumors.

We demonstrate that STAT3 and EMT pathways are over-activated in progenitor-like BTSCs. STAT3 is a direct transcription factor for TWIST [37], a key regulator of SNAIL [38] ZEB1 and ZEB2 [39]. However, we show that SLUG is the key EMT regulator in progenitor-like BTSCs. While SLUG was recently shown to be a direct STAT3 target [40], we add to this finding that SLUG is the only EMT transcription factor directly regulated by STAT3 in BTSCs. SLUG enhances the migratory and invasive abilities of BTSCs both in vitro and in vivo. Surprisingly, while no obvious differences were seen in vitro, SLUG overexpression dramatically enhanced proliferation in vivo, resulting in significantly shorter survival. The divergence in the results obtained in vitro and in vivo suggest that the microenvironment may contribute to the pro-proliferative role of SLUG. Moreover, SLUG overexpression in a quiescent stem-like BTSC line resulted in tumor initiation and dramatically increased tumor burden. These data support the premise that engrafted stem-like BTSCs retain a relatively quiescent state; requiring a STAT3-driven, SLUG-mediated shift toward a progenitor precursor state to become “activated” and more tumorigenic. We also observed an activated inflammatory environment in SLUG_OE tumors reminiscent of mesenchymal GBMs. This suggests that SLUG and the BTSC precursor state may be involved in this pro-inflammatory phenotype typically associated with mesenchymal GBMs. Interestingly, progenitor-like BTSCs overexpress cytokines such as IL6, IL8, CCL2, and TGFβ (not shown), which may promote recruitment and activation of innate immune cells. Of note, such cytokines are classical upstream signaling factors in EMT that are often triggered upon injury and inflammation. Investigating the upstream signaling that promotes a STAT3/SLUG mediated precursor state transition will shed further light on whether the tumor microenvironment, maybe in response to the injury/inflammation caused by the treatment, may promote such transition and provide insight for future therapeutic opportunities. Finally, our experimentally-derived SLUG_OE score was associated with recurrence in our BTSC cohort, in a pair of primary/recurrent tumors and derived BTSCs and in the TCGA GBM cohort. Taken together, these findings strongly suggest that the STAT3/SLUG-driven precursor state transition toward a more aggressive progenitor-like state is central to the rise of post-treatment recurrence in GBM. However, we fully expect this precursor state transition to be complex and involve factors in addition to SLUG. The Wnt/β-Catenin pathway, for example, regulates stemness as well as cell fate determination and is central to EMT [41,42]. Interestingly, the Wnt/β-catenin pathway cross-talks with the STAT3 pathway [43], is modulated by E-cadherin and regulates EMT transcription factors such as SLUG and TWIST [44,45]. Moreover, while not playing a direct role in the regulation of the BTSC precursor state under our culture conditions, key EMT transcription factors other than SLUG likely play important roles in tumor progression by contributing to essential processes such as invasion and survival and proliferation. Interestingly, ZEB1, the most expressed EMT regulator across our BTSC lines, plays central roles in regulating differentiation and invasion in both NSCs during development and glioma cells [46,47].

Despite our findings, the clinical relevance of BTSC precursor states remains elusive. BTSCs are isolated from patient tumors and cultured in conditions with high levels of EGF, FGF, and often LIF [18,20], all of which are potent STAT3 activators. These artificial in vitro culture conditions may contribute to the activation of the STAT3-driven EMT signaling. BTSCs largely maintain their phenotype in vitro across passages. However, before a BTSC line is considered “established,” patient-derived primary cells are often grown for extended periods of time. This is possibly a sensitive period during which the precursor state of BTSCs may be modulated until a specific equilibrium is reached in vitro. While challenging, investigating these initial putative changes in BTSC precursor state upon BTSC establishment may be particularly interesting. We expect all BTSC lines to contain both stem-like and progenitor-like cells in order to maintain a proper hierarchy and drive propagation both in vitro and when xenografted. However, the balance between stem-like and progenitor-like cells that is eventually reached in different BTSC lines, may be quite variable and may explain the heterogeneity found across BTSCs. Importantly, while culture conditions likely affect the precursor state of BTSCs, these distinct states may be pre-existing in the tumor. In future studies, it will be essential to confirm that stem-like and progenitor-like populations are present in patients’ tumors and whether they are enriched in newly-diagnosed primary GBMs and recurrent GBMs, respectively. Interestingly, while we focus here on two distinct precursor states, the STAT3-driven EMT process may regulate progression through a spectrum of states ranging from proneural/stem-like to mesenchymal/progenitor-like, which may better explain the inter- and intra-heterogeneity found in BTSCs and GBMs. Moreover, while we argue here that the STAT3/SLUG-driven precursor state shift is at the root of the PMT, it may not be restricted only to proneural and mesenchymal GBMs. Rather than a full switch from proneural toward the mesenchymal subtype, a subtle mesenchymal shift in GBM from different transcriptomic subtypes may better encompass recurrences initiated through a STAT3/SLUG-driven precursor state shift in the CSC compartment upon treatment. Bulk and single cell RNA-sequencing data from matching pairs of primary/recurrent tumors and BTSCs will be required to investigate these critical questions and understand the processes underlying progression and recurrence in GBM.

Collectively, our results suggest that a mesenchymal shift at recurrence originates from a STAT3/SLUG-driven precursor state transition, within the stem cell compartment of the tumor and provide new perspectives on the mechanisms driving GBM recurrence. Related findings from other groups help support the premise that activation of the STAT3/SLUG axis and subsequent precursor state transition is radiation-induced [6,7,40]. While not the focus of the present study, we and others have shown the efficacy of several clinically relevant inhibitors targeting the STAT3 pathway in BTSCs both in vitro and in vivo [21,30,31]. Specifically, inhibiting the STAT3/SLUG pathway, using such inhibitors concurrent with standard of care treatment could prevent a therapy-induced stem-like to progenitor-like transition and prevent or delay recurrence. Interestingly, EMT is a reversible process [48] and, while SLUG knock-down/knock-out experiments are needed to explore the reversibility of the BTSC precursor state transition, one may reason that therapeutic intervention could reverse the aggressive phenotype of progenitor-like BTSCs toward the less aggressive, more quiescent stem-like state. Thus, therapeutic strategies preventing a precursor state transition in the CSC compartment, by inhibiting the STAT3/SLUG pathway, may lead to better tumor control and significantly prolong survival in GBM.

## 4. Materials and Methods

### 4.1. BTSC Lines

We performed transcriptomic analysis on a subset of 57 BTSC lines (listed in Figure 1B) representative of a larger collection of over 150 BTSC lines established from patient samples in our laboratory. This heterogenous subset of BTSC lines was initially selected for a comprehensive genomic profiling effort of matched glioblastoma tumors, cell-lines, and xenografts [49]. The selection of the BTSCs was based on material availability as well as clinical, genomic, and transcriptomic data, to best represent the molecular alterations classically found in GBM. This cohort contains, in similar proportions, BTSC lines derived from proneural, classical, and mesenchymal GBMs. For in vitro experiments, we used several BTSC lines to validate our results. RT-qPCR analysis of the expression of SLUG following inhibition of the STAT3 pathway (R333, Figure 3A) was performed on 12 BTSC lines (BT12, BT25, BT50, BT53, BT67, BT69, BT73, BT75, BT85, BT124, BT134, BT147). Assessment, at the protein level, of STAT3 activity and SLUG levels in progenitor-like BTSCs and stem-like BTSCs (Figure 2B) was performed on a subset of eight BTSC lines (BT12, BT50, BT73, BT89, BT124, BT134, BT147, BT206). Assessment, at the protein level, of STAT3 activity and SLUG levels in progenitor-like and stem-like BTSCs in absence of growth factors or when supplemented with EGF was performed on two stem-like and two progenitor-like BTSC line (BT75, BT73, BT67, and BT69) (Figure S5E). These western blots (Figure 2B and Figure S5E) shows some of the more stereotypical stem-like and progenitor-like BTSCs and were selected based on their stem-like to progenitor-like score and phenotypical characteristics [24]. It is important to note that while this is a “small” validating subset, overexpression of SLUG and STAT3 activity in progenitor-like BTSC has been confirmed at the transcript level across all 57 cells lines. STAT3 ChIP-PCR of BTSCs grown under standard culture conditions (Figure 3H) was performed on a representative set of six BTSC lines (BT50, BT67, BT69, BT89, BT94, BT147). Similarly, whole genome ChIP-sequencing (Figure 3I) was performed of this same set of BTSC lines (BT50, BT67, BT69, BT89, BT94, BT147). Overexpression of SLUG and evaluation of its impact on growth and migration in vitro was performed on a total of five BTSC lines (BT50, BT67, BT69, BT124, BT147). Representative BTSC lines were used (as described in the legends) when they were the most relevant for specific experiments. For example, BT67 was used for most of the in vitro assessment of the impact of inhibition and activation of STAT3 on SLUG expression as it displays average levels of activated STAT3 and presents good responses to both activation and inhibition of the pathway (Figure 3B,C and Figure S5). Similarly, BT67 was used for the ChIP-PCR upon OSM-mediated STAT3 activation (Figure 3G). In contrast, BT124 was used specifically for overexpression of STAT3 (Figure 3D) as it has one of the lowest basal levels of STAT3 expression across our BTSC lines and was thus the best candidate to overexpress STAT3 and cSTAT3. For in vitro and in vivo experiments, upon SLUG overexpression (Figure 4 and Figure 5), BT69 was chosen as it is the BTSC line that has the lowest basal expression of SLUG. Moreover, it was also the BTSC line with the lowest migration potential of the BTSCs tested in chemotaxis migration assay in vitro. Finally, BT50 was specifically used to study the impact of SLUG overexpression on tumor growth in vivo because it is our most stereotypical stem-like BTSCs (Figure 6). Moreover, based on historical data, BT50 displays a mostly quiescent phenotype when implanted in vivo as it does not generate large tumors but live cells can still be detected a year post-xenograft. BT50 was thus an ideal choice to test whether SLUG overexpression in stem-like BTSCs promotes a more progenitor-like, more tumorigenic phenotype in vivo. Finally, we used our only primary/recurrent pair of tumor tissue (189T/248T) and the matching BTSC lines that we successfully established (BT189/BT248), to examine the transcriptomic changes arising upon recurrence.

### 4.2. Cell Culture

BTSC lines were cultured as previously described [20]. AlamarBlue (Life Technologies, (Grand Island, NY, USA) was used to monitor growth and viability. Sphere size was monitored over time with an IncuCyte (Essen Bioscience, Ann Arbor, MI, USA). For limiting dilution assay (LDA), cells were plated by serial dilutions from 1024 cells down to 1 cell per well (8 wells/dilution). LDAs were performed in triplicate, scored after 2 weeks and analyzed with ELDA. ELDA (extreme limiting dilution assay [50]) is a software application for limiting dilution analysis (LDA), with particular attention to the needs of stem cell assays. A command-line version of ELDA is available In R through the limiting function of the statmod package. Alternatively, a webtool (http://bioinf.wehi.edu.au/software/elda/) also provides an easy user interface.

STAT3 pathway inhibition was performed with two JAK2 inhibitors (R333 [21] and SB1518 [51]) and two direct STAT3 inhibitors (SH-04-54 [31] and STATTIC [32]). Our laboratory has extensively investigated targeting the STAT3 pathway in BTSCs in previous studies [21,30,31,51]. Notably, STAT3 inhibition concurrent with current standard of care was shown to be an effective therapeutic strategy to target BTSCs. Short treatments (4–24 h) were performed to evaluate the impact of STAT3 pathway inhibition on SLUG. Historical data from our group and the literature have previously shown that short-term STAT3 inhibition has limited cytotoxicity on BTSCs. Longer-time points, where both cytotoxic and cytostatic effects can be observed, were used to evaluate the impact of STAT3 inhibition on migration (72 h) and viability (14 days) in SLUG_OE BT69, are compared to CTRL BT69.

### 4.3. Immunoblotting

BTSC pellets (as detailed in Section 4.1 and Figure legends) were lysed in RIPA buffer (50 mM Tris, 150 mM NaCl, 0.1% SDS, 0.5% Na deoxycholate, and 1% NP40) and Complete Protease Inhibitor Cocktail Tablets (Roche, Indianapolis, IN, USA). A total of 20 μg of protein was separated by SDS-PAGE and transferred onto a nitrocellulose membrane according to standard protocols. Blots were blocked in 5% non-fat milk TBS and incubated overnight with primary antibody at 4 °C followed by a one-hour incubation with the appropriate horseradish peroxidase-conjugated secondary antibody. pSTAT3 Y705 (1:1000, CST, #9145S), STAT3 (1:1000, SCBT, SC-8019), SLUG (1:750, SCBT, SC-166476), Actin (1:2000; SCBT, SC-1615), Tubulin (1:4000, CST, #2146S), and horseradish-conjugated donkey anti-mouse, donkey anti-rabbit and donkey anti-goat (1:5000, Millipore). Quantification was performed using the gels tools in ImageJ.

### 4.4. Real-Time Quantitative PCR

RNA was extracted using the RNeasy Plus kit and reverse transcribed using the Sensiscript RT (Qiagen). qPCR was performed using the FastStart essential PCR Mastermix on a lightcycler 96 (Roche).

*ACTIN*: F: *CATGTACGTTGCTATCCAGGC*; R: *CTCCTTAATGTCACGCACGAT*
*SNAI2*: F: *TCGGACCCACACATTACCTTG*; R: *AAAAAGGCTTCTCCCCCGTGT*
*CDH1*: F: *CCCAATACATCTCCCTTCACAG*; R: *CCACCTCTAAGGCCATCTTTG*

### 4.5. Transcriptomic Data

RNA sequencing was performed as previously described [24] on 57 BTSC lines, a representative subset of our collection of 150 patient derived BTSC. RNA-seq pair-end reads were aligned to a transcriptome reference consisting of the reference genome extended by the annotated exon-exon junctions [52]. To generate transcriptome reference we used the JAGuaR v 1.7.6 pipeline [53], specifically developed to allow the possibility for a single read to span multiple exons. Reads aligned to a custom transcriptome reference (build from NCBI GRCh37-lite reference and Ensembl v69 gene annotations) are then “repositioned” on to genomic coordinates, transforming reads spanning exon-exon junctions into large-gapped alignment. Using repositioned reads, the RPKM (Reads Per Kilobase per Million) metric [54] was calculated for every collapsed transcripts gene model that we used in the subsequent analysis. Collapsed transcripts gene model was defined by overlapping of all exons of all known isoforms for a given gene. Expression data on the 57 BTSC lines was made publicly available on the European bioinformatic institute data sharing platform, EGA (European Genome-phenome Archive, Accession number: EGAS00001002709).

For RNA-sequencing of BT50 CTRL and SLUG_OE, RNA was extracted from BT50 CTRL and SLUG_OE cell pellets (n = 3) using the RNAeasy kit from Qiagen. Samples were prepared with Illumina’s TruSeq Stranded mRNA Library preparation kit. The final libraries were validated with the Agilent 2200 TapeStation D1000 assay and quantitated by Kapa qPCR Library Quantification Kit for Illumina. The sequencing was performed on the Illumina NextSeq 500 instrument. RNA-Seq reads were then pseudoaligned to the human NCBI RefSeq transcript database (January 2017), using Kallisto 0.42.4.

RNA-sequencing of our primary/recurrent pair of tumor tissue (189T/248T) and the matching BTSC lines (BT189/BT248), that we successfully established, was performed as described below. RNA from tissue and cells were extracted using Qiagen AllPrep DNA/RNA/miRNA Universal Kit (Cat. 80224). Strand-specific RNA-seq (ssRNA-seq) library were constructed from total RNA samples using polyA capture of transcripts. Libraries were sequenced on Illumina HiSeq2500 machine (indexed lane using V4 chemistry) generating 75bp paired reads. Reads were aligned with the STAR aligner v2.4.2a to hg38 human reference genome (from iGenome). R Bioconductor DESEq2 package was used for normalization and vst transformation of the gene expression matrix.

GBM tumor samples analysis is based on transcriptomic data from The Cancer Genome Atlas program of the national cancer institute (TCGA). GBM transcriptomic data from either a large TCGA set of 539 primary GBM samples (Affymetrix HT Human Genome U133a microarray platform) for Kaplan-Meier analysis or a smaller, RNA sequenced, TCGA set of 172 samples (IlluminaHiSeq) that includes some recurrent GBMs, as indicated in the legends. The gene expression profile was measured experimentally using the Affymetrix HT Human Genome U133a microarray platform by the Broad Institute of MIT and Harvard University cancer genomic characterization center or the Illumina HiSeq 2000 RNA Sequencing platform by the University of North Carolina TCGA genome characterization center, respectively. In both cases, level-3-interpreted level data were downloaded from TCGA data coordination center (version: 2015-02-24). All the clinical data used to analyze survival and annotate samples as normal tissues, primary or recurrent GBM samples as well as information regarding GBM subtype can be found along with the transcriptomic data on the TCGA data coordination center (https://portal.gdc.cancer.gov/projects/TCGA-GBM).

### 4.6. Signature Scores

Through cluster analysis of NSC characteristics such as self-renewal, stem and progenitor marker expression, quiescence, and asymmetric cell division, which were evaluated in 20 BTSC lines, we previously identified distinct stem-like and progenitor-like BTSCs, predictive of survival after xenograft. We also identified 136 differentially expressed genes in stem-like and progenitor like BTSCs [24]. The stem-like to progenitor-like score represents the sum of the z-scores of genes previously found to be upregulated in progenitor-like BTSCs minus the sum of the z-scores of genes upregulated in stem-like BTSC lines (Table S1) [24]. Briefly, we first calculated z-scores, across the whole dataset, for each of the 136 differentially expressed genes between stem-like and progenitor-like BTSCs listed in Table S1:

$$z=\frac{X-\mu }{\sigma }$$

where x is the level of expression, µ is the average expression across all samples and σ the standard deviation, for a given gene. We then calculated the sum of the z-scores of genes overexpressed in stem like BTSCs (z’[stem-like genes]) and the sum of the z-scores for genes overexpressed in progenitor-like BTSCs (z’[progenitor-like genes]). We further calculated the stem-like to progenitor-like score = z’[progenitor-like genes] − z’[stem-like genes]. The higher the score, the more similar is a sample’s expression profile to the progenitor-like profile. Therefore, this score ranks samples (Figure 1B) from stem-like to progenitor-like as defined in [24].

The proneural to mesenchymal score is the sum of the z-scores of 198 mesenchymal genes minus the sum of the z-score of 151 proneural genes. The proneural and mesenchymal genes are the genes used to define proneural and mesenchymal GBMs in [2] and are listed in Table S1. We first calculated the z-scores, across the whole dataset for each of the mesenchymal and proneural genes as described above. We then calculated the sum of the z-scores of mesenchymal genes (z’[mesenchymal genes]) and the sum of the z-scores of proneural genes (z’[proneural genes]). We then calculated the proneural to mesenchymal score, which is equal to z’[mesenchymal genes] − z’[proneural genes]. The higher the score, the more similar is a sample’s expression profile to the mesenchymal profile. Therefore, this score ranks samples from proneural to mesenchymal as defined in [2].”

The STAT3 score represents the sum of the z-scores of 57 validated STAT3 target genes reviewed in [55] and listed in Table S1. We first calculated z-scores, across the whole dataset for each of the validated STAT3 targets as described above. We then calculated the sum of the z-scores of these STAT3 target genes (z’[STAT3 target genes]). Therefore, this STAT3 score ranks samples according to the overall expression of STAT3 transcriptional targets which is directly representative of STAT3 activity.

The EMT score is based on a published multi-cancer metastasis-associated signature containing genes coordinately overexpressed only in a subset of malignant samples that have exceeded a particular staging threshold specific to each cancer type and reflecting an invasive transition. This signature, highly enriched in EMT markers, was used as a mesenchymal transition signature and found to be relevant to all solid tumor types including non-epithelial cancers such as neuroblastomas, Ewing’s sarcoma, and GBMs [11,56]. In the present study, z-scores were calculated across the whole dataset for each of the 64 mesenchymal transition associated genes from the multi-cancer metastasis-associated signature described in [56] and listed in Table S1. The EMT score represents the sum of these 64 z-scores. The higher the score, the more is a sample’s expression profile associated to a multi-cancer mesenchymal transition profile [11,56].

The SLUG_OE score is the sum of the z-score of upregulated genes upon SLUG overexpression in BT50 minus the sum of the z-score of downregulated genes. Briefly, we first calculated the z-scores, across the whole dataset for each of the differentially expressed genes. We then calculated the sum of the z-scores of upregulated genes upon SLUG overexpression (z’[SLUG upregulated genes]) and the sum of the z-scores of downregulated genes upon SLUG overexpression (z’[SLUG downregulated genes]). We then calculated the SLUG_OE score, which is equal to z’[SLUG upregulated genes] − z’[SLUG downregulated genes]. The higher the score, the more similar is a sample’s expression profile to the changes triggered by the overexpression of SLUG in BT50, a stem-like BTSC line.

### 4.7. Differential Genes Expression Analysis and Gene Ontology Analysis

Sleuth was used for differential gene expression analysis upon SLUG overexpression (BT50) Transcripts passing the Wald test (FDR < 0.05) were considered differentially expressed. GORILLA [57] and REViGO [58] were used for Gene Ontology analysis.

### 4.8. Overexpression of STAT3 and SLUG in BTSCs

Wild-type STAT3 (Sino Biological, Wayne, PA, USA) and a constitutively active STAT3 (EF.STAT3C.Ubc.GFP, Addgene, Watertown, MA, USA) were cloned into a constitutive Piggy Back vector (PB513B-1, System Biosciences). Empty, STAT3WT and STAT3C PiggyBack constructs were electroporated in BT124 along with the Super PB transposase (PB210PA-1, System Biosciences) using the Neon electroporation system (3 pulses, 20 ms, 1200 mv). Cells were submitted to puromycin selection 7 days after electroporation.

For SLUG overexpression, BTSCs were transduced with lentiviral particles produced in HEK293T transfected (lipofectamine 2000, Invitrogen, Carlsbad, CA, USA) with a second-generation lentivirus packaging system (pMD2.G and pCMV delta R8.2, Addgene) and control or SLUG transfer vector (PS100064 and RC202365L1, Origene, Rockville, MD, USA). Media of transfected HEK293T cells was replaced 24 h post-transfection and then collected every 24 h for 3 days. Lentiviral particles were concentrated from the collected media using lenti-X concentrator (Clontech, # 631231, Mountain View, CA, USA) according to manufacturer’s instructions. A piggybac/transposon expression system was also used. SLUG (pPGS-hSLUG.fl.flag, Plasmid #25696) was cloned into a cumate inducible piggy back vector (PBQM812A-1, System Biosciences, Palo Alto, CA, USA). Empty and SLUG constructs were electroporated along with the Super PB transposase (PB210PA-1, System Biosciences, Palo Alto, CA, USA) using the Neon electroporation system (3 pulses, 20 ms, 1200 mv). Cells were submitted to puromycin selection 7 days after electroporation. This system was used to overexpress SLUG in BT67, BT69, BT124, and BT147 in Figure S6 and validate the results obtained with lentiviral-mediated overexpression of SLUG in BT50 and BT69, which are otherwise used throughout the manuscript.

### 4.9. Chromatin Immunoprecipitation (ChIP)

A total of 10 million BTSCs (as detailed in Section 4.1 and Figure legends) were treated with OSM, maintained in SFM (−EGF/FGF) or (+EGF/FGF) for 6 h. ChIP was performed using the ChIP-IT High Sensitivity kit (Active Motif). Immunoprecipitations were performed on 10 ug of sheared chromatin with either 2 ug Phospho-Stat3-Tyr705 (CST) or 2 ug of Normal Rabbit IgG (CST). Primers were designed to amplify a 180 pb DNA fragment containing a putative STAT3 binding site found in the SLUG (SNAI2) promoter just upstream of the TSS identified using TFBSearch. Positive control primer sets were designed to amplify DNA fragment containing validated STAT3 binding site in the promoters of GFAP and STAT3. Negative control primer sets (Active Motif, #71001) were also used.

STAT3: F: 5′CTCCCTGAGTTGGCTGTTCT3′; R: 5′GAGCCGTATCAGGGCATTTA3′
GFAP: F: 5′TCCGAGAAGCCCATTGAG3′; R: 5′TGTGCTGCTTTTATCCCAAGA3′
SLUG: F: 5′GCACCACATAAAAGCAGGGG3′; R: 5′GGGGGCAAGAGGTAACTGTC3′

For ChIP-sequencing, 15 ng of immunoprecipitated and input DNA were used for library preparation and sequenced on an Illumina NextSeq 500 with 150 cycle mid-output. Raw reads were aligned against the human genome version hg19.

### 4.10. Migration and Invasion

Migration experiments were performed using 96-Well Chemotaxis Plates in an IncuCyte (Essen Bioscience, Ann Arbor, MI, USA). Top and bottom membranes were coated with collagen I. 2500 BTSCs (as detailed in Section 4.1 and Figure legends) were seeded in the top chamber in SFM (+EGF/FGF). The bottom chamber was supplemented with 1% FBS as a chemoattractant. Pictures focused on both the upper and the bottom sides of the membrane are taken over time. For visualization purposes, these images can be overlaid highlighting cells on the upper and bottom sides of the membrane with distinct colors. The Essen Bioscience chemotaxis software can measure cell area over time on the top (cells that did not migrate) or the bottom (cells that did migrate) sides of the membrane. Cell area on the bottom side of the membrane was used to quantify migration over time. For invasion, ~100 µm BTSC spheres (BT69) were seeded in collagen I and cell area was monitored over time in the IncuCyte. To quantify invasion from the embedded spheres, cell area was normalized to the area of the spheres at T0.

### 4.11. Intracranial BTSC Xenografts

All animal procedures were performed according to our animal ethics protocol (AC17-0215), approved by the Animal Care Committee of the University of Calgary and operating under the Guidelines of the Canadian Council on Animal Care. All attempts are made to minimize the handling time during surgery and treatment so as not to unduly stress the animals. At the time of cell implantation animals are anesthetized intraperitoneally with a mixture of ketamine and xylazine. Animals receive buprenorphrine immediately prior to and following surgery to relieve pain. Animals are observed daily after surgery to ensure there are no unexpected complications. In the extremely rare case that an animal is observed to be in significant pain or distress following surgery (weight loss <20%, lack of grooming, lethargy, vocalization, etc.,), it was euthanized immediately by overdose of Euthanyl.

For in vivo invasion assay, 50,000 cells (BT69) were implanted in the corpus callosum of C17/SCID mice (n = 3). Coordinates for stereotactic implantation were as follows: anteroposterior −1.0, mediolateral 2.0, and dorsoventral 1.7. Four weeks post-implantation, mice were euthanized by overdose of Euthanyl and brains were harvested, sectioned, and stained with anti-human nucleolin antibody. To quantify invasion, hNucl positive cells were counted within the invasion zones (2 rectangles on Figure 3H) and normalized to the hNucl positive area of the whole brain section.

For all BTSC lines orthotopically xenografted for survival studies (detailed in Section 4.1, figures and legends), 100,000 cells were implanted in the right striatum of C17/SCID mice. Coordinates for stereotactic implantation were as follows: anteroposterior −1.0, mediolateral 2.0, and dorsoventral 3.0. Mice were sacrificed by overdose of Euthanyl upon significant weight loss or presentation of neurologic symptoms necessitating euthanasia.

### 4.12. MRI

Animals were monitored in the Experimental Imaging Center (University of Calgary, Calgary, Alberta, Canada). T2 weighted sequence with cryocoil, (TE 48 ms) was performed using a 9.4-T Bruker horizontal-bore magnetic resonance (MR) system. MRI was used to detect any lesion (enhancing or not) that could potentially represent tumor growth, edema, tumors necrosis/hematomas. Dashed lines on images (Figure 6A) loosely delineates areas of abnormal enhancement that potentially represent tumor growth. MRI was strictly used to define the earlier time point at which animals should be euthanized for further histologic analysis. Interpretations as to the presence of a tumor was based solely on histology and hNucl staining.

### 4.13. Immunohistochemistry

Heat-induced antigen retrieval with sodium citrate was performed on formalin-fixed OCT cryo-sections according to standard protocols. Anti-human nucleolin antibody (1:1000, Abcam, # AB13541, Cambridge, UK), Ki67 (1:250, Vector labs, VP-K451) and Iba1 (1:500, Waco, #019-19741) were used and ABC Elite kit (Vector Labs, Burlingame, CA, USA) and diaminobenzidine (Sigma, St Louis, MI, USA) were used to develop the staining with hematoxylin counterstaining.

### 4.14. Statistics

Pearson correlation, two-tailed T-tests, one-way and two-way ANOVAs were performed as appropriate. Log-rank test was used in Kaplan-Meier survival analysis.

## 5. Conclusions

We propose that the rapid emergence of lethal post-treatment recurrences in glioblastoma may be the result of a shift toward a more progenitor-like precursor state in the cancer stem cell compartment post-treatment. Our findings demonstrate a STAT3-driven activation of an EMT-like process mediated by SLUG in progenitor-like BTSC. SLUG drives a shift from a stem-like to progenitor-like precursor state, thus promoting a more aggressive, more tumorigenic phenotype. Blocking this transition would maintain brain tumor stem cells in a more quiescent stem-like precursor state and, in turn, delay the emergence of fatal recurrences characteristic of glioblastoma. Our work provides a strong rationale for developing therapeutic strategies targeting the STAT3 pathway, concurrently with standard of care treatment, to block this transition and improve survival.

## Supplementary Materials

The following are available online at https://www.mdpi.com/2072-6694/11/11/1635/s1, Figure S1: Stem- and progenitor-like precursor state associate respectively with proneural and mesenchymal GBMs. Figure S2: Activated STAT3 and EMT pathways associated with mesenchymal GBMs. Figure S3: STAT3 and EMT pathways are associated with BTSC precursor states. Figure S4: Correlations of expression of EMT transcription factors with STAT3 and EMT pathways activity. Figure S5: SLUG is a direct target of STAT3. Figure S6: SLUG overexpression promotes migration but does not impact growth in vitro. Figure S7: SLUG overexpression does not affect BTSC sphere forming capacity, EGF/FGF dependency, or sensitivity to STAT3 inhibition. Figure S8: STAT3/SLUG-driven transition toward a progenitor-like precursor state underlies progression to recurrence in a matched pair of primary/recurrent tumors and derived BTSCs. Table S1: List of genes used to calculate the stem-like to progenitor-like, proneural to mesenchymal, EMT and STAT3 scores. Table S2: Gene Ontology analysis (BT50).
