# Supplementary material for: SLUG Directs the Precursor State of Human Brain Tumor Stem Cells
Source: Cancers (Basel). 2019 Oct 24;11(11):1635. doi: 10.3390/cancers11111635 (PMC6895861; doi:10.3390/cancers11111635)
Supplement: Supplementary file 1 [file cancers-11-01635-s001.zip › cancers-610664-supplement-final.docx]

Supplementary Material

SLUG Directs the Precursor State of Human Brain Tumor Stem Cells

**Table S1.** List of genes used to calculate the stem-like to progenitor-like, proneural to mesenchymal, EMT and STAT3 signature scores. List of overexpressed genes in stem-like and progenitor-like BTSCs [24], proneural/mesenchymal genes [2], validated STAT3 transcriptional target genes [55] and EMT related genes [56]. * Uploaded separately as excel table.

**Table S2.** Gene Ontology analysis (BT50). List of significant GO terms showing enrichment upon SLUG overexpression in BT50. * Uploaded separately as excel table.


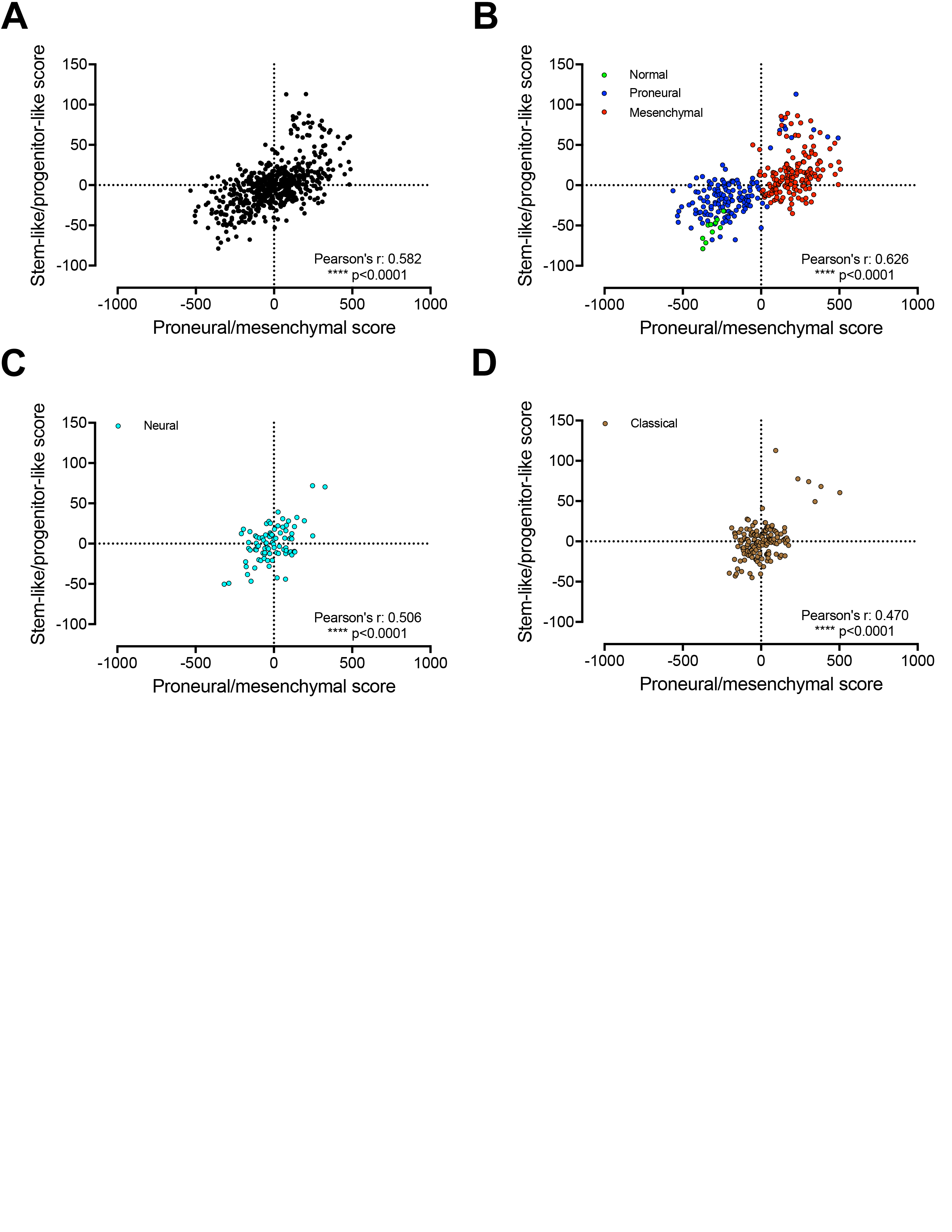


**Figure S1.** Stem- and progenitor-like precursor state associate respectively with proneural and mesenchymal GBMs. (**A**) Scatter plot of the correlation between the stem-like to progenitor-like score and the proneural to mesenchymal score in GBM samples from a TCGA cohort of over 500 GBM samples and including normal brain tissue samples (Affymetrix U133a microarray platform). Scatter plot of the stem-like to progenitor-like score and the proneural to mesenchymal score restricted to (**B**) normal brain tissue samples, proneural and mesenchymal GBMs, or including only (**C**) neural or (**D**) classical GBMs.


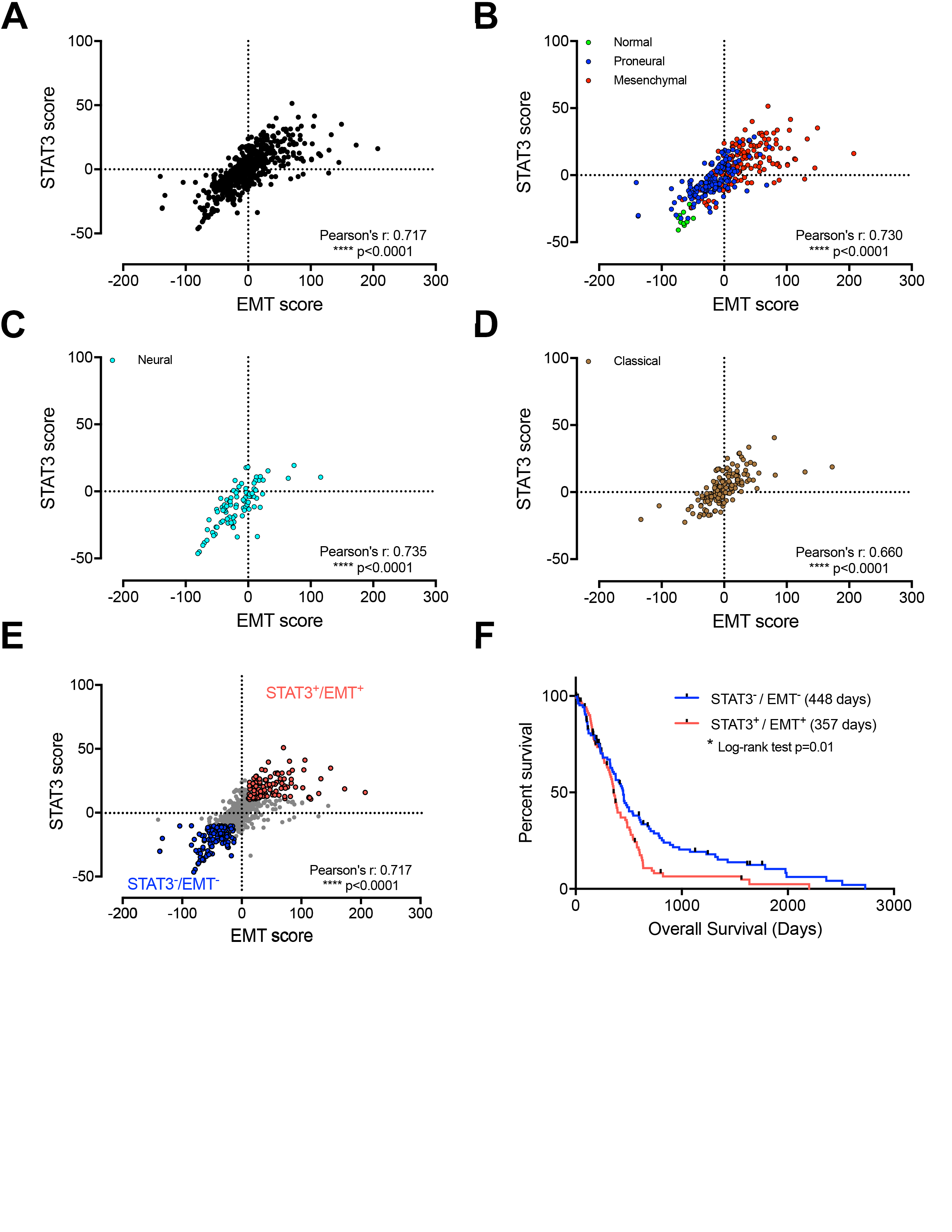


**Figure S2.** Activated STAT3 and EMT pathways associate with mesenchymal GBMs. (**A**) Scatter plot of the correlation between STAT3 and EMT scores in GBM samples from a TCGA cohort of over 500 GBM samples and including normal brain tissue samples (Affymetrix U133a microarray platform). Scatter plot of STAT3 and EMT scores restricted to (**B**) normal brain tissue samples, proneural and mesenchymal GBMs, or including only (**C**) neural or (**D**) classical GBMs. (**E**) Scatter plots representing the correlation between STAT3 and EMT scores amongst TCGA all GBM samples and highlighting STAT3^+^/EMT^+^ (red, 104 samples) and STAT3^-^/EMT^-^ (blue, 105 samples) samples (−10< z-score>10) and (**F**) Kaplan Meier curves for these subsets showing significantly shorter survival of STAT3^+^/EMT^+^ GBM patients.

**
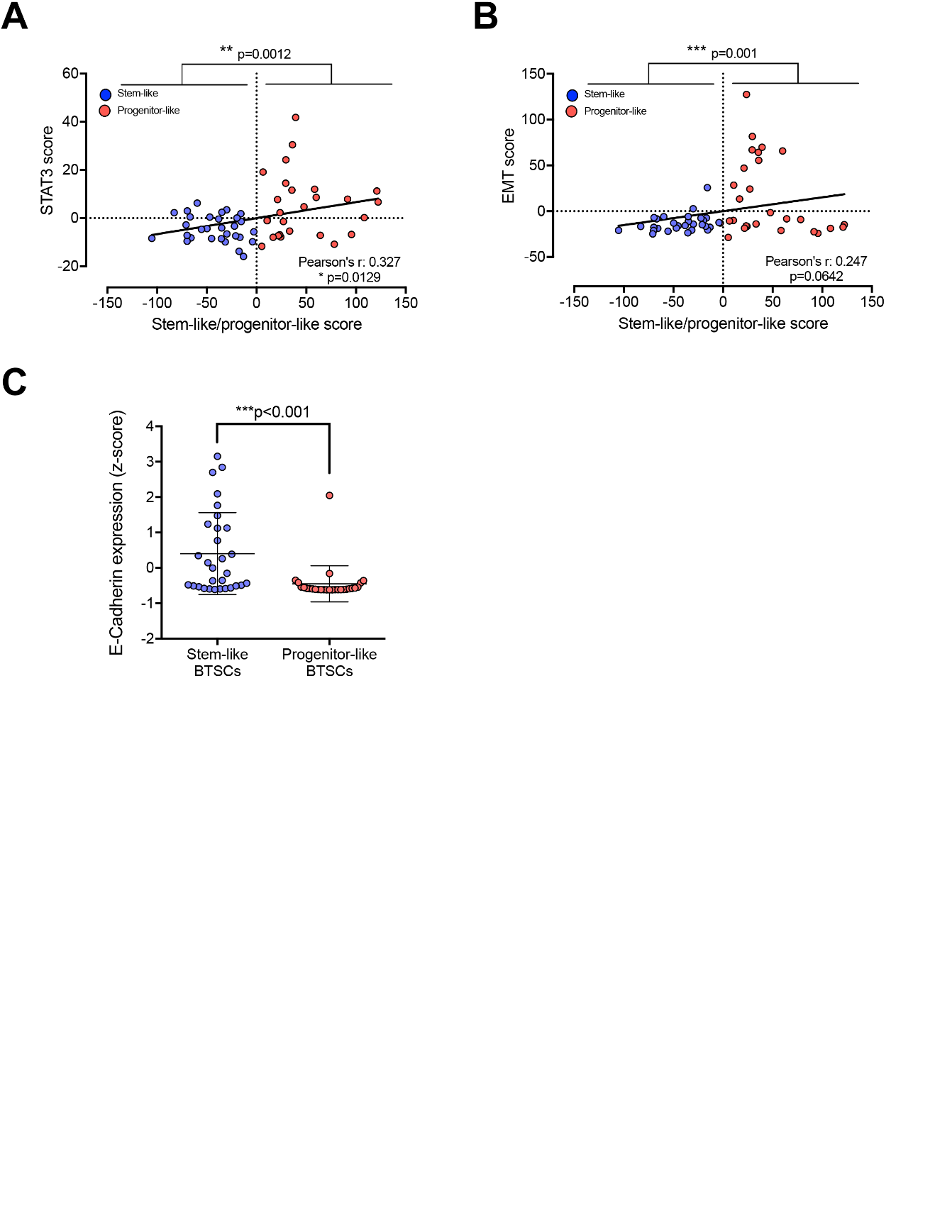
**

**Figure S3.** STAT3 and EMT pathways are associated with BTSC precursor states. Scatter plots showing the correlation between the stem-like to progenitor-like score and (**A**) STAT3 and (**B**) EMT scores in BTSCs. T-tests were also performed to test for statistical difference in STAT3 and EMT scores between stem-like and progenitor-like BTSCs. (**C**) Graphical representation of E-cadherin expression in Stem-like and progenitor-like BTSCs. Figures are based on RNA-sequencing performed on 57 BTSC lines.

**
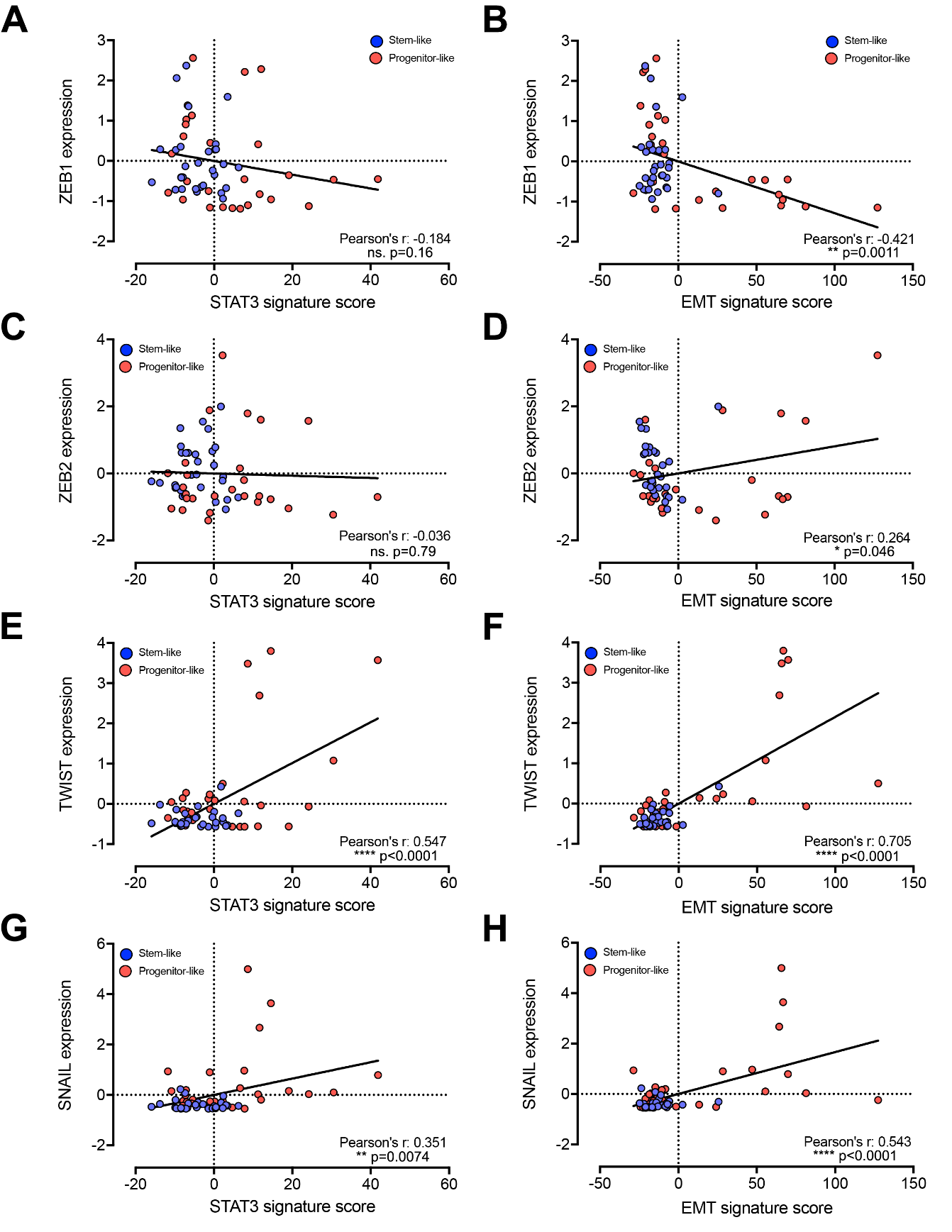
**

**Figure S4.** Correlations of expression of EMT transcription factors with STAT3 and EMT pathways activity. Scatter plots showing the correlation between the expression of ZEB1 (**A** and **B**), ZEB2 (**C** and **D**), TWIST (**E** and **F**), and SNAIL (**G** and **H**) with STAT3 and EMT scores in BTSCs segregated as stem-like or progenitor-like. All figures are based on RNA-sequencing performed on 57 BTSC lines.


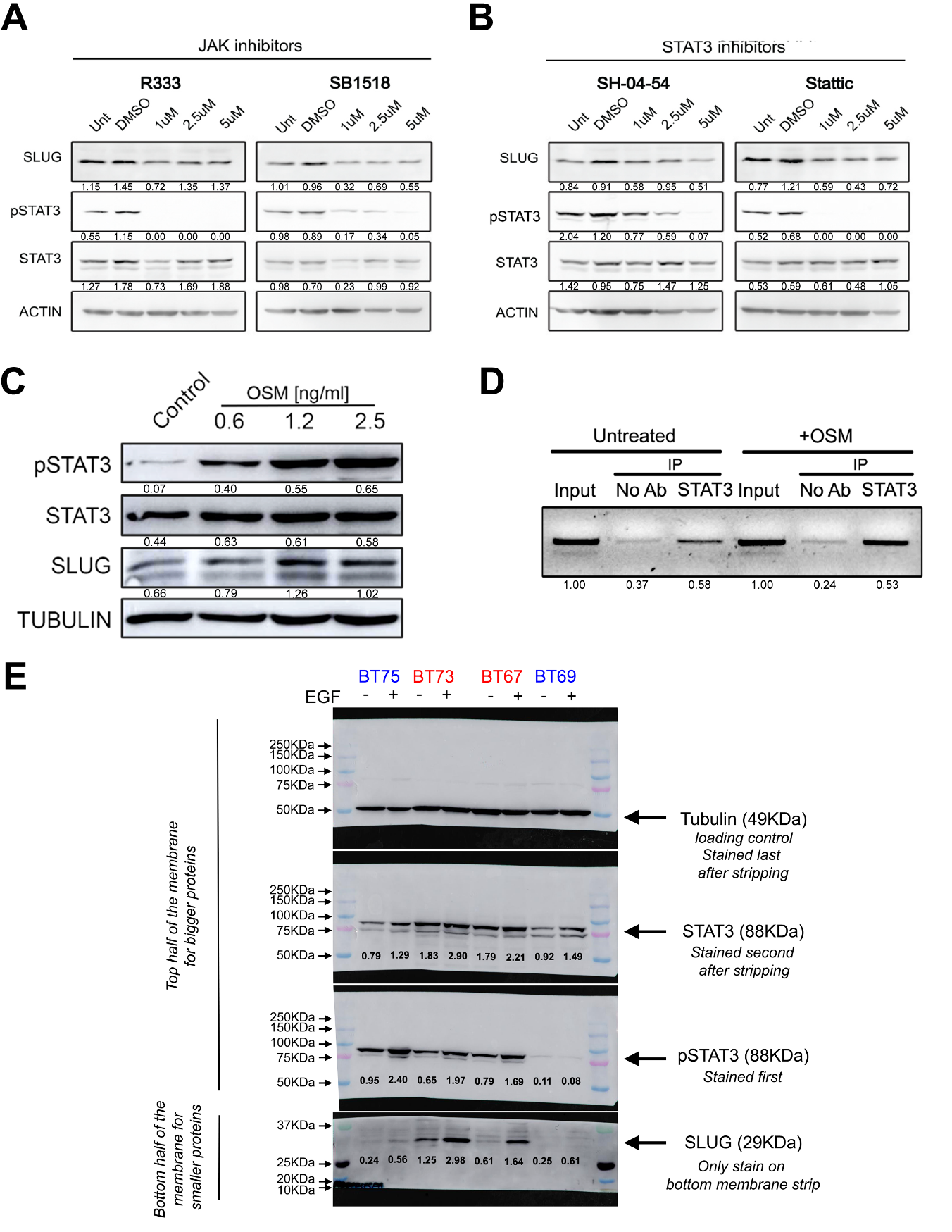


**Figure S5.** SLUG is a direct target of STAT3. Representative western blots of BTSCs treated with (**A**) JAK2 inhibitors (R333 and SB1518) and (**B**) direct STAT3 inhibitors (SH-04-54 and STATTIC), and the impact of treatment on pSTAT3 (Y705) and SLUG. (**C**) Representative western blot showing activation of STAT3 pathway and increased SLUG levels following OSM treatment and (**D**) DNA gel showing increased STAT3 binding to the SLUG promoter upon OSM treatment. (**E**) Western blot showing full membrane and size marker for validation of the antibodies used in the study (STAT3 phospho-tyrosine STAT3 and SLUG) in stem-like (blue) and progenitor-like (red) BTSCs grown in absence of growth factors or supplemented with 20 ng/mL EGF. Quantification of the western blot is relative to loading control and relative to input in the DNA gel.


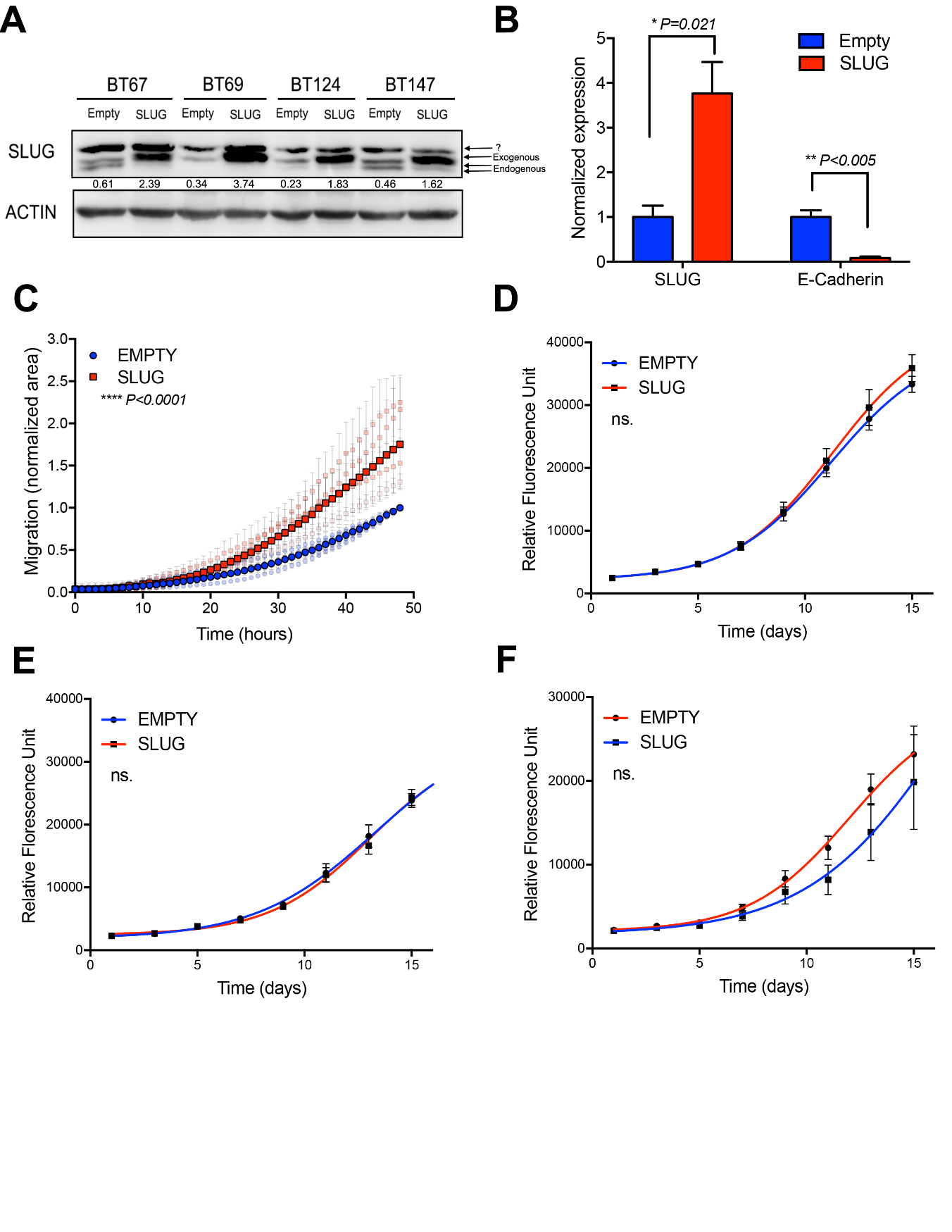


**Figure S6.** SLUG overexpression promotes migration but does not impact growth in vitro*.* (**A**) Western blot showing SLUG overexpression and (**B**) RT-qPCR data showing increased SLUG expression and decreased E-cadherin expression in 4 BTSC lines (BT67, BT69, BT124 and BT147). (**C**) Graphical representation of the significant migration increase assay upon SLUG overexpression in BTSC lines (BT67, BT69, BT124 and BT147). Main curves represent migration data pooled from the 4 BTSC lines and lighter curves the individual BTSC lines. Growth curves (Alamar blue assay) showing no significant impact of SLUG overexpression on growth in (**D**) BT147, (**E**) BT67 and (**F**) BT124.


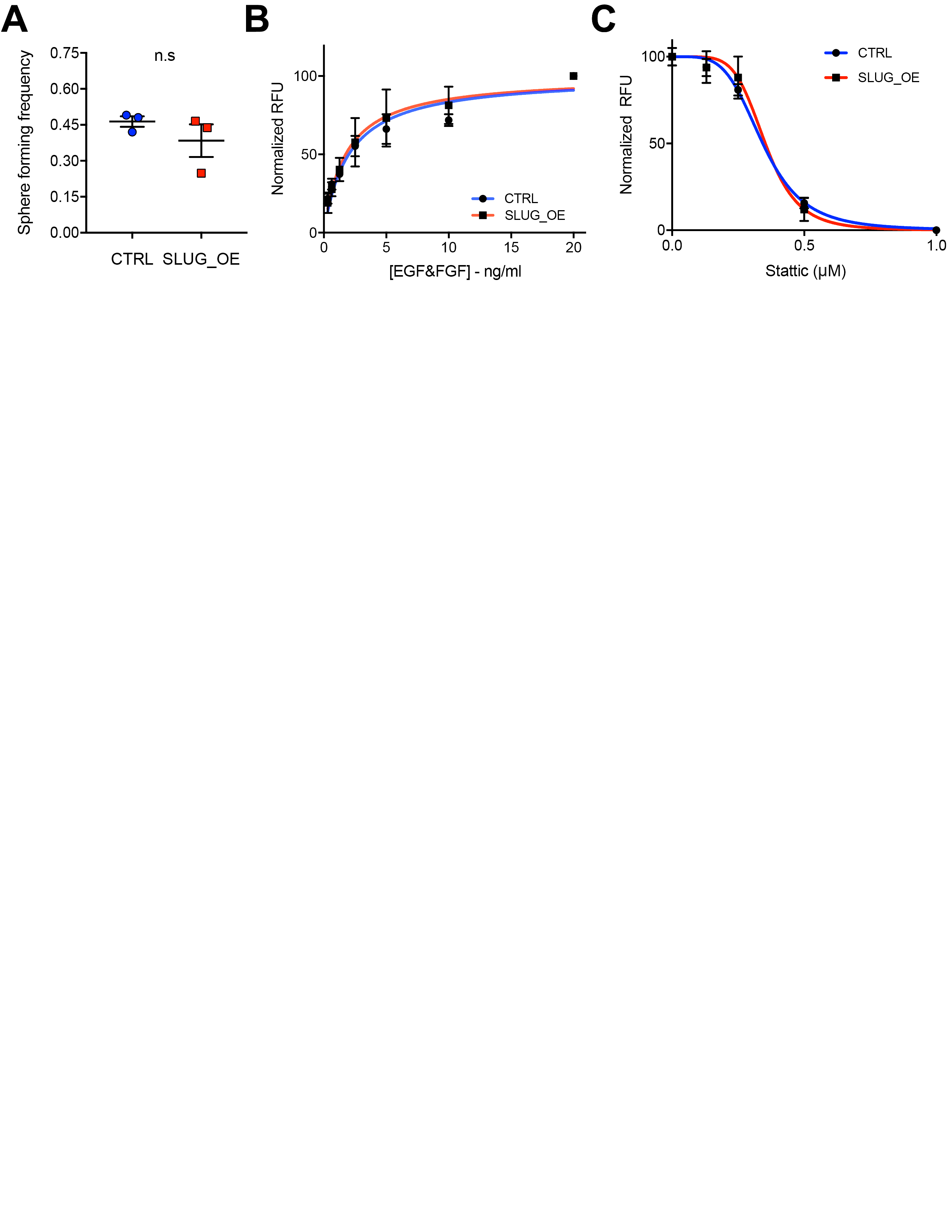


**Figure S7.** SLUG overexpression does not affect BTSC sphere forming capacity, EGF/FGF dependency or sensitivity to STAT3 inhibition. Graphs representing (A) BT69 CTRL and SLUG_OE sphere forming frequency (limiting dilution analysis, LDA), (B) growth dependency on EGF and FGF and (C) sensitivity to STAT3 inhibition with STATTIC (error bars represent SEM).


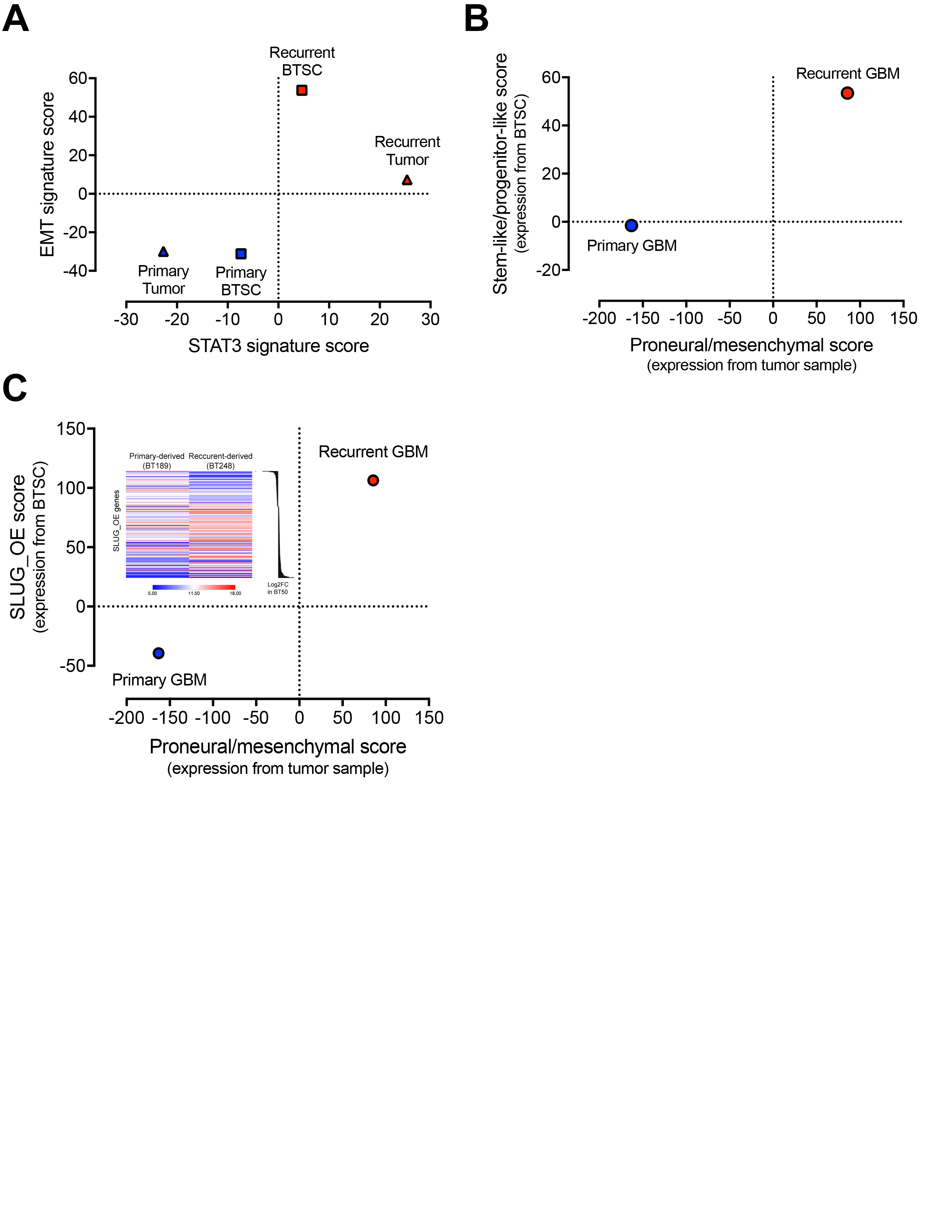


**Figure S8.** STAT3/SLUG-driven transition toward a progenitor-like precursor state underlies progression to recurrence in a matched pair of primary/recurrent tumors and derived BTSCs. (**A**) Scatter plot showing increased STAT3 and EMT scores upon recurrence in both tumor samples (T189/T248) and derived BTSCs (BT189/BT248). (**B**) Scatter plot showing increased proneural to mesenchymal score in the tumor (T189/T248) and stem-like to progenitor-like score in the matched BTSC (BT189/BT248) upon recurrence. (C) Scatter plot of the proneural to mesenchymal score in tumor samples (T189/T248) versus the SLUG_OE score of the matched BTSC lines (BT189/BT248). Heatmap (insert) represents the expression of the 180 genes of the SLUG_OE score in BT189 and BT248.
